# Estrogen alpha receptor antagonists for the treatment of breast cancer: a review

**DOI:** 10.1186/s13065-018-0472-8

**Published:** 2018-10-25

**Authors:** Deepika Sharma, Sanjiv Kumar, Balasubramanian Narasimhan

**Affiliations:** Faculty of Pharmaceutical Sciences, Maharshi Dayanand University, Rohtak, Haryana 124001 India

**Keywords:** Estrogen receptor alpha, Antiestrogens, Relative binding affinity, Molecular docking, Breast cancer

## Abstract

**Background:**

Cancer is at present one of the leading causes of death in the world. It accounts for 13% of deaths occurred worldwide and is continuously rising, with an estimated million of deaths up to 2030. Due to poor availability of prevention, diagnosis and treatment of breast cancer, the rate of mortality is at alarming level globally. In women, hormone-dependent estrogen receptor positive (ER+) breast cancer making up approximately 75% of all breast cancers. Hence, it has drawn the extensive attention of researchers towards the development of effective drugs for the treatment of hormone-dependent breast cancer. Estrogen, a female sex hormone has a vital role in the initiation and progression of breast malignancy. Therefore, estrogen receptor is the central target for the treatment of breast cancer.

**Conclusion:**

In this review, we have studied various classes of antiestrogens that have been designed and synthesized with selective binding for estrogen alpha receptor (ER). Since estrogen receptor α is mainly responsible for the breast cancer initiation and progression, therefore there is need of promising strategies for the design and synthesis of new therapeutic ligands which selectively bind to estrogen alpha receptor and inhibit estrogen dependent proliferative activity.

## Background

### Global scenario of breast cancer

According to breast cancer statistics obtained from the global cancer project (GLOBOCAN, 2012), it was observed that 5,21,907 approx deaths cases recorded worldwide in 2012 were due to breast cancer. With the increase in age, the risk for breast cancer and death rates due to it generally increases [1]. The highest incidence of breast cancer was in Northern America and Oceania and the lowest incidence in Asia and Africa. In non-Hispanic white (NHW) and non-Hispanic black (NHB) women the frequency of occurrence and death due to breast cancer are higher than other racial groups. Global differences in the rates of breast cancer are affected by changes in risk factors prevalence and poor diagnosis of it. Adaptation of western lifestyle [2, 3] and delayed childbearing [4, 5] has increased the risk of breast cancer among Asian and Asian American women [2]. The extent of events of breast cancer increases among Hispanic and Hispanic American women especially due to delayed childbearing [2]. In contrast, African countries show approximately 8% new cases of breast cancer; most of the deaths occur due to the limited treatment and late stage diagnosis. According to World Health Organization (WHO 2015) reports, the highest incidence rates of breast cancer were recorded in Malaysia and Thailand [6]. In light of above, in the present review we have covered the role of estrogen receptor α antagonists as anticancer agents against breast cancer especially over the past decade as there was no such extensive report is found in the literature.

### Role of estrogen alpha in breast cancer

Estrogen, a female sex hormone, related physiological functions are exhibited mostly by the estrogen receptors subtypes’ ER-*α* and *β*. The estrogen receptor alpha has leading role in uterus and the mammary gland. Aromatase enzyme synthesizes 17*β*–estradiol from andostenindione. This synthesized estradiol (E2) binds to the estrogen receptor which is located in the cytoplasm undergoes receptor dimerization and this estradiol-ER complex translocated into the nucleus where this complex further bind to DNA at specific binding sites (estrogen response element). In response to estradiol hormone binding, multiprotein complexes having coregulators assemble and activate ER− mediated transcriptional activity via ER designated activation functions AF1 and AF2 to carry out the estrogenic effects. The deregulation in the functioning of these various coregulators such as alteration in concentration of coregulators or genetic dysfunctionality leads to uncontrolled cellular proliferation which results into breast cancer. Such as loss of the epithelial adhesion molecule Ecadherin leads to metastasis by disrupting intercellular contacts. Deregulation of MTA1 coregulator, enhances transcriptional repression of ER, resulting in metastasis. The AIB1 (ER*α* coregulator) get amplified, results in the activation of PEA3-mediated matrix metalloproteinase 2 (MMP2) and MMP9 expression which cause metastatic progression. Another ER coregulator SRC-1, has promoted breast cancer invasiveness and metastasis by coactivating PEA3-mediated Twist expression. In recent study, PELP1 overexpression results into ER*α*- positive metastasis. Collectively, these studies showed that ERα coregulators modified expression of genes involved in metastasis [7, 8].

### Mechanism of action of estrogen alpha receptor antagonists

Endocrine therapy is first choice treatment for the most of the ER+ve breast cancer patients. Currently, three classes of endocrine therapies are widely used.

**Fig. 1:**
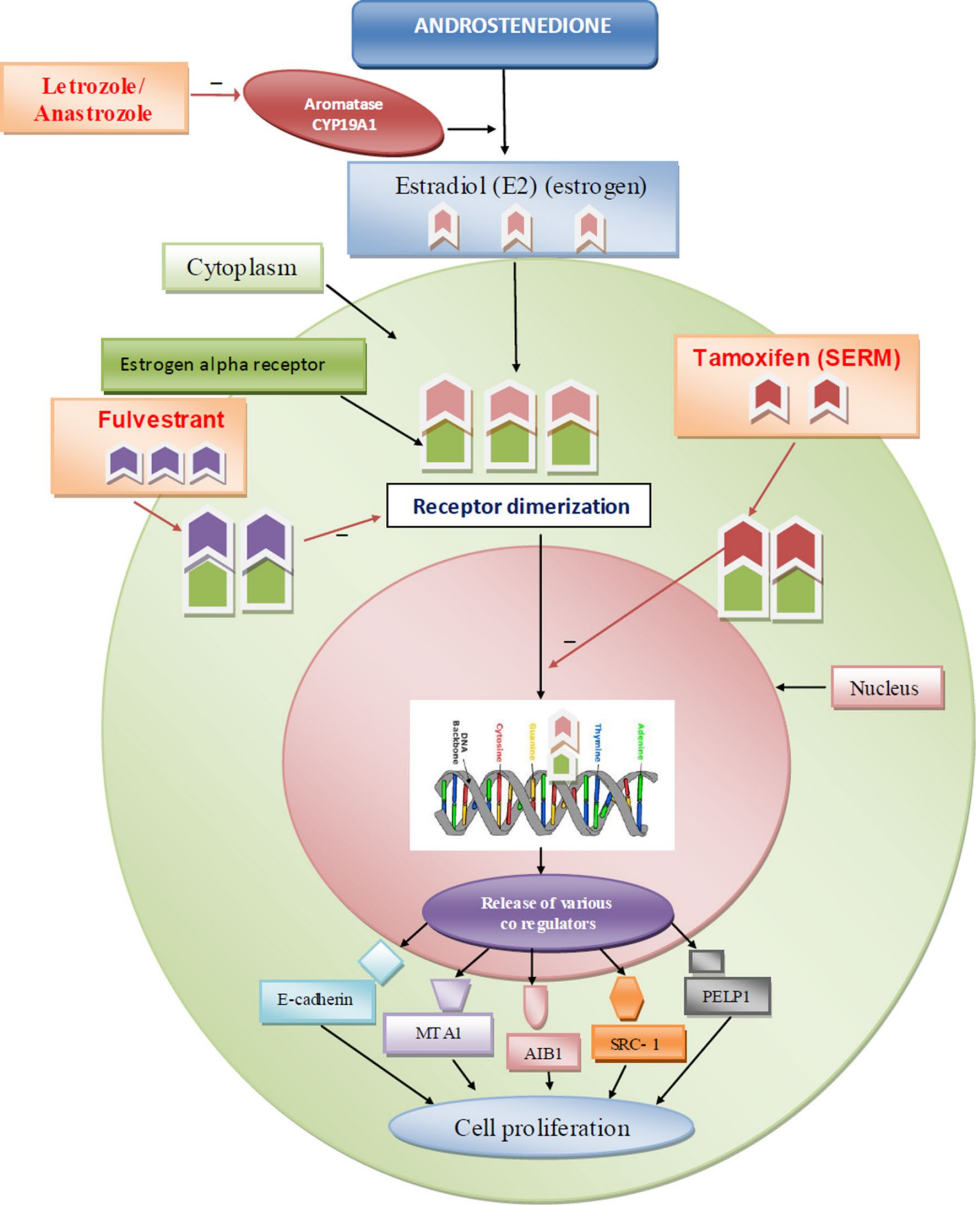
Role of estrogen alpha receptor and estrogen alpha receptor antagonists (tamoxifen, fulvestant, letrozole and anastrozole) in breast cancer

- Aromatase inhibitors (AIs): Letrozole and anastrozole decrease the estrogen production by inhibiting the aromatase enzyme thus suppressing the circulating level of estrogen [8].
- Selective estrogen receptor down regulators (SERDs): Fulvestrant, competitively inhibits estradiol binding to the ER, with greater binding affinity than estradiol. Fulvestrant–ER binding impairs receptor dimerisation, and energy-dependent nucleo-cytoplasmic shuttling, thus blocking nuclear localisation of the receptor [9].
- Selective estrogen modulator: Tamoxifen competitively bind with the estrogen receptor and displaces estrogen and thus inhibits estrogen function in breast cells. The co-activators are not binding but, inhibiting the activation of genes that enhance cell proliferation [8]. The flow diagram of role of estrogen receptor and estrogen receptor antagonist is as shown in Fig. 1.

Efforts have been aided for estrogen receptor subtype-selectivity by making changes in the structural configuration of estrogen receptors to develop specific ER− pharmacophore models. The newly developed antiestrogens should not only have good binding affinity with particular receptor but it also must have selective activation for that receptor which expressed in breast cancer progression. Therefore, selective ER *α* antagonists may be helpful for the breast cancer treatment [10].

### Rationale of study

**Fig. 2:**
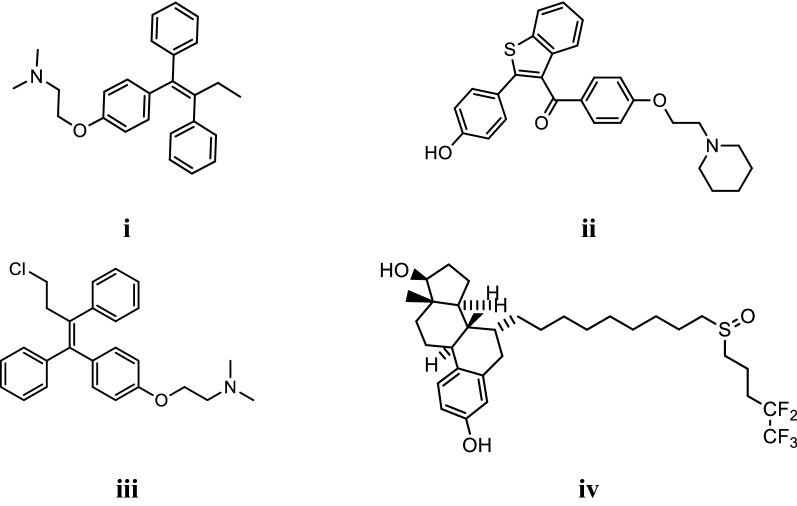
Marketed drugs for breast cancer

Currently, a number of breast cancer drugs are available in Fig. 2 [11, 12] namely: tamoxifen (i), raloxifene (ii), toremifene (iii) and fulvestrant (iv) but they have following limitations:

I. Tamoxifen is the drug of choice to treat patients with estrogen related (ER) breast tumors. Resistance to tamoxifen develops after some years of treatment due to change in its biocharacter from antagonist to agonist and it is also responsible for the genesis of endometrial cancer [9].
II. Women who take toremifene for a longer period to treat breast cancer are at higher risk of development of endometrial cancer.
III. Raloxifene an oral selective estrogen receptor modulator increases the incidence of blood clots, deep thrombosis and pulmonary embolism when taken by breast cancer patients.
IV. Fulvestrant down regulates the ER α but it has poor pharmacokinetic properties i.e. low solubility in water.

### Various heterocyclic analogues as estrogen alpha receptor antagonists

#### Dibenzo[b, f]thiepines analogues

**Fig. 3:**
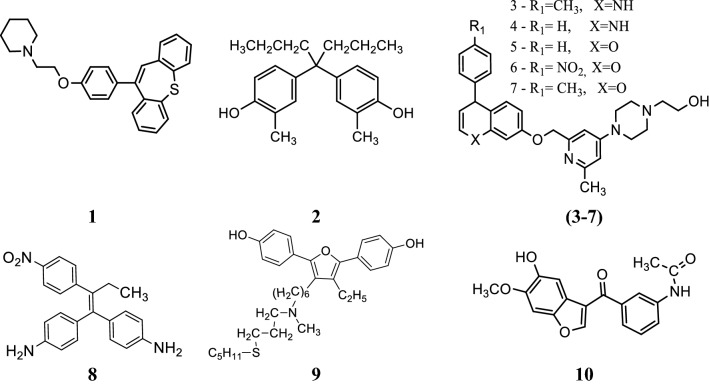
Molecular structures of compounds (**1**–**10**)

**Fig. 4:**
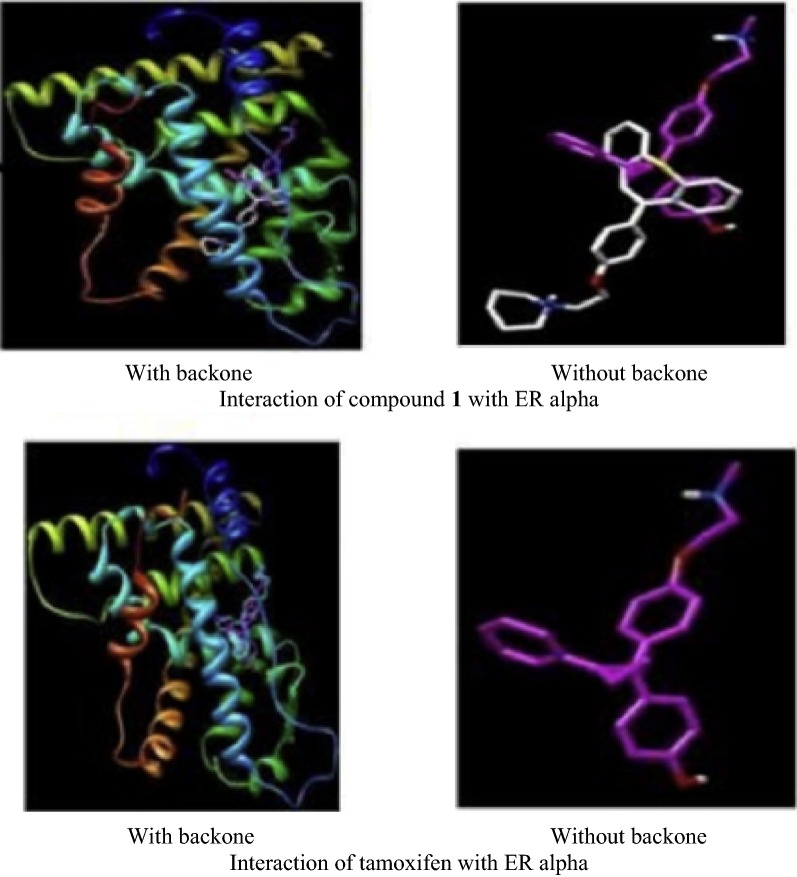
Pictorial presentation of interaction of compound **1** and tamoxifen with ER alpha

Ansari et al. [13], developed some molecules of dibenzo[*b,f*]thiepine and evaluated their antiproliferative potential against ER + ve (MCF-7) cancer cell line using MTT assay. Among synthesized derivatives, compound **1**, (Fig. 3)] exhibited the potent anticancer activity with IC_50_ value 1.33 µM against MCF-7 tumor cell line, due to arrest in G0/G1phase of cell cycle. Molecular docking studies carried out by MGL Tools 1.5.4 revealed that the tricyclic core of the compound **1** occupied the same binding space in the ER-α pocket as tamoxifen. The most active compound **1** showed significant homology with tamoxifen while interacting with amino acids (GLY390, ILE386, LEU387, LEU391, LEU403, GLU353, LYS449 and ILE326) of ER-*α* but the basic side chain (3^o^ amino alkoxy) orientated opposite to that of tamoxifen (Fig. 4). Thus, it showed that compound **1** exhibited the better binding affinity with ER alpha as compared to tamoxifen (9.6 ± 2.2 µM) and this improved binding might be responsible for good anti-estrogenic potential.

### Diphenylmethane skelon

Maruyama et al. [14], synthesized some derivatives of diphenylmethane as estrogen antagonist that would bind to the estrogen receptor similar as estradiol. The antagonistic activity of synthesized derivatives was evaluated by AR reporter gene assay. Among the synthesized compounds, compound **2**, [4,4′-(heptane-4,4-diyl)bis(2-methylphenol) (Fig. 3)] was found to be potent one and displayed 28-times more selectivity for estrogen receptor alpha (IC_50_ = 4.9 nM) over estrogen receptor beta (IC_50_ = 140 nM). The binding interactions of compound **2** were determined computationally using AutoDock 4.2 program into ER-*α* (PDB ID: 3UUC). Docking study showed that phenol group of compound **2** interacted with the amino acid E353 of ER-*α* through H-bonding and the bulky side chain (*n*-Propyl) present at the central carbon atom of bisphenol A directed towards the amino acid M421 of ER-*α*.

**Fig. 5:**
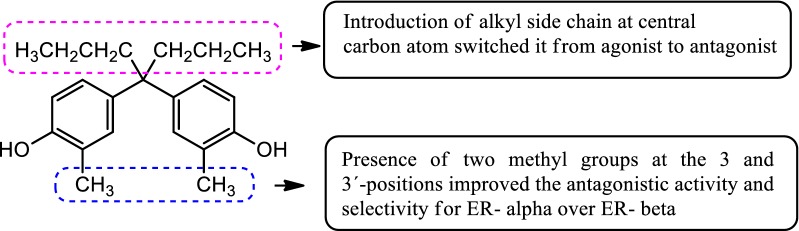
Structure activity relationship study of compound **2**

SAR: Thus, introduction of alkyl chains at central carbon atom switched it from agonist to antagonist and presence of two methyl groups at the 3 and 3′-positions improved the antagonistic activity and selectivity for ER-*α* over ER-*β* (Fig. 5).

### Conjugated heterocyclic scaffolds

**Fig. 6:**
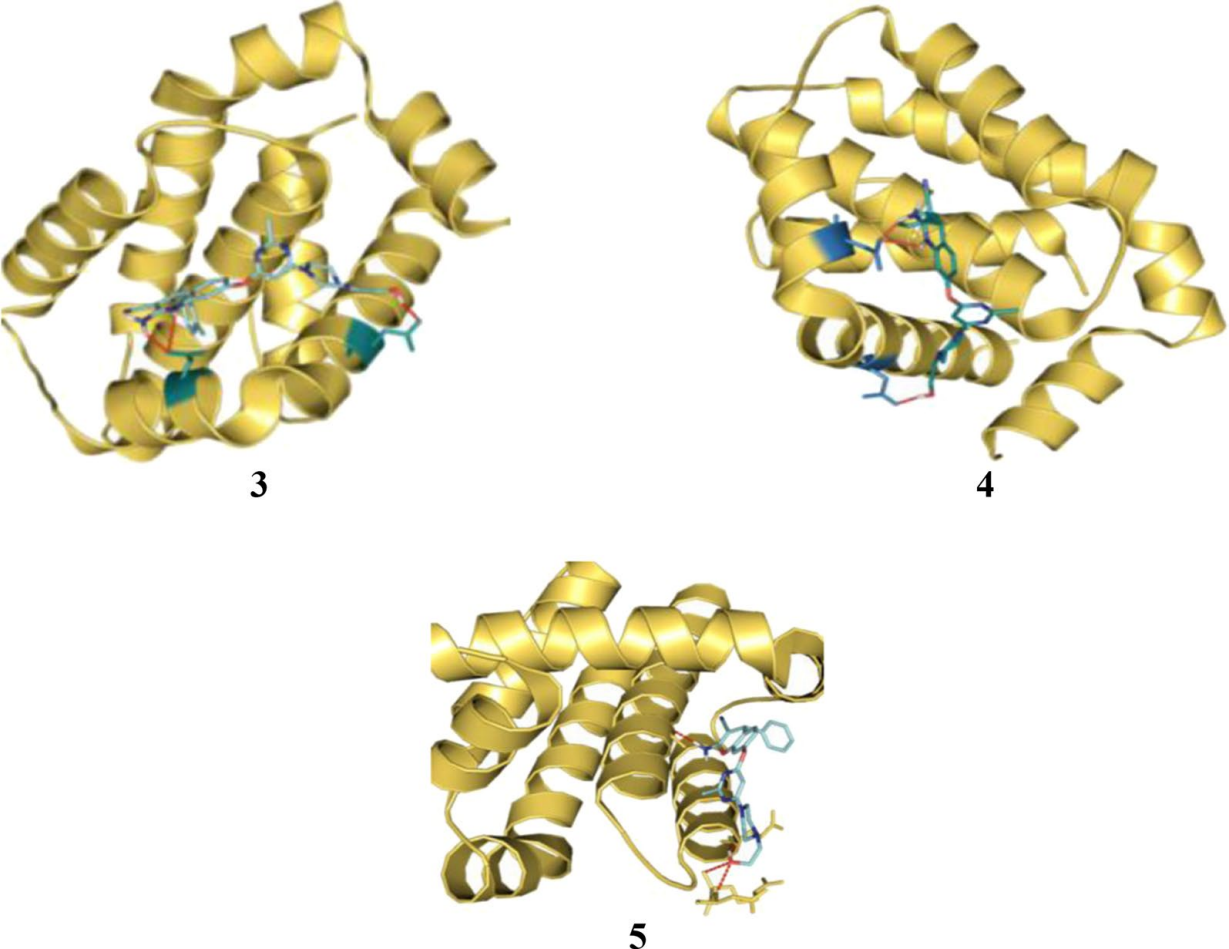
Pictorial presentation of best conformation of compounds **3**–**5**

Parveen et al. [15], developed new conjugates of pyrimidine-piperazine, chromene and quinoline. Antiproliferative activity of the synthesized conjugates was determined against (MCF-7) tumor cell line using MTT assay. Among these conjugates, compound **3**, (2-(4-(2-methyl-6-((4-*p*-tolyl-1,4-dihydroquinolin-7-yloxy)methyl)pyridin-4-yl)piperazin-1-yl) ethanol), 4, (2-(4-(2-methyl-6-((4-phenyl-1,4-dihydroquinolin-7-yloxy)methyl)pyridin-4-yl) piperazin-1-yl ethanol), 5, (2-(4-(2-methyl-6-((4-phenyl-4*H*-chromen-7-yloxy)methyl) pyridin-4-yl)piperazin-1-yl)ethanol), 6, (2-(4-(2-methyl-6-((4-(4-nitrophenyl)-4*H*-chromen-7-yloxy)methyl)pyridin-4-yl)piperazin-1-yl) ethanol) and 7, (2-(4-(2-methyl-6-((4-*p*-tolyl-4*H*-chromen-7-yloxy)methyl)pyridin-4-yl)piperazin-1-yl)ethanol) showed good anti-proliferative activities as compared to standard curcumin (Table 1, Fig. 3). Molecular docking of most active compounds **3**, **4** and **5** against 3D structure of Bcl-2 protein was performed using Autodock 4.2 (Fig. 6). The Lamarckian genetic algorithm (LGA) was applied to study the protein-ligands interactions. The *p*-tolyl present in compound **3** and phenyl group present in compound **4** formed three hydrogen bond one with amino acid Asp100 and two with amino acid Asp108 respectively. The chromene ring in compound **5** formed four hydrogen bond with Glu133, Ala146, Arg136 and Asp137 with good binding interaction having binding energy (∆G) − 7.70 kcal/mol, Ki = 2.26 µM). The most favorable binding within the active sites of BCL-2 was shown by compounds **3** and **4** with minimum binding energy (∆G) = − 9.08 kcal/mol and (∆G) = − 8.29 kcal/mol, respectively.

**Table 1:** Anticancer activity (IC_50_ = µM) results of conjugates **3**–**7**

Compound No.	Cancer cell line MCF-7
**3**	48 ± 1.70
**4**	65 ± 1.13
**5**	92 ± 1.18
**6**	30 ± 1.17
**7**	16 ± 1.10
Curcumin	48 ± 1.11

SAR: Structure–activity relationship study showed that the anticancer potential improved when chromene and quinoline nucleus combined with piperazine and pyrimidine rings.

### Aromatase inhibitors/selective estrogen receptor modulator

**Fig. 7:**
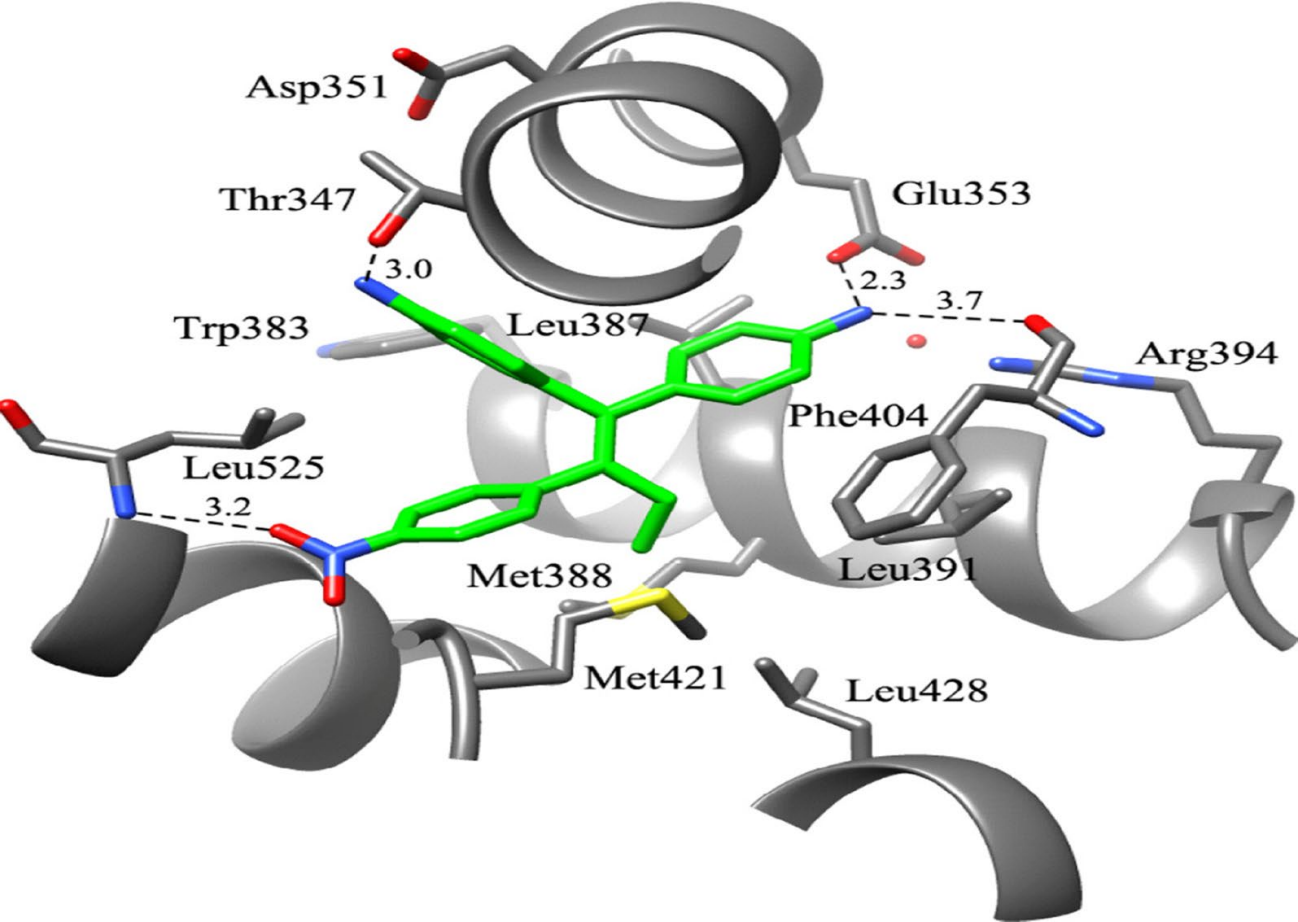
Docking model of compound **8**

Zhao et al. [16], designed and synthesized selective estrogen receptor modulators (SERMs) based on diphenylmethylene scaffold by incorporating some of the structural features of the aromatase inhibitor letrozole into lead compound (norendoxifen) by bis-Suzuki coupling to generate a series of selective anti-breast cancer agents to address the problem of *E, Z* isomerization related with norendoxifen. The functional cellular assay method was employed on MCF-7 cancer cells to evaluate the aromatase inhibitory potential indicated that compound **8**, (Fig. 3) was the most active one (IC_50_ = 62.2 nM). The binding pattern of the most active one (**8**) was determined using docking software GOLD3.0 In compound **8**, the amino substituent present on the phenyl ring that is cis conformation to the nitrophenyl nucleus formed H- bond with the OH group of Thr347 while the other amino substituent formed H-bond to the carboxylate of amino acid Glu353 and the backbone bonded to the carbonyl of Phe404 of ER-*α* (PDB-3ERT) as shown in Fig. 7. The binding affinity of compound **8** for both ER-*α* and ER-*β* was found to be (EC_50_ = 72.1 nM) and (EC_50_ = 70.8 nM), respectively.

### Furan derivatives

Zimmermann et al. [17], prepared estrogen antagonists by incorporating side chains having amino or sulfur functional groups linked at 3rd position of furan for the breast cancer therapy. The synthesized furan derivatives were determined for their anticancer potential against MCF-7/2a breast cancer cells line. The degree of alpha selectivity increased from 2.5 to 236 times when alkyl group attached at 4th position of furan nucleus. Especially, compound **9**, (4,4′-(3-ethyl-4-(6-(methyl(3-(pentylthio)propyl)amino)hexyl)furan-2,5-diyl) diphenol showed the strongest antiestrogenic effect (Table 2, Fig. 3). It was found that 2,5-bis(4-hydroxyphenyl)furans with two short alkyl chains have better binding interactions with ER *α* than that for ER *β*.

**Table 2:** Antiestrogenic and antiproliferative activity of compound 9

Compound No.	(IC_50_ = µM)	
	Antiestrogenic activity	Antiproliferative activity (MCF-7)
**9**	0.050	0.022
Fulvestrant	0.003	0.004

**Fig. 8:**
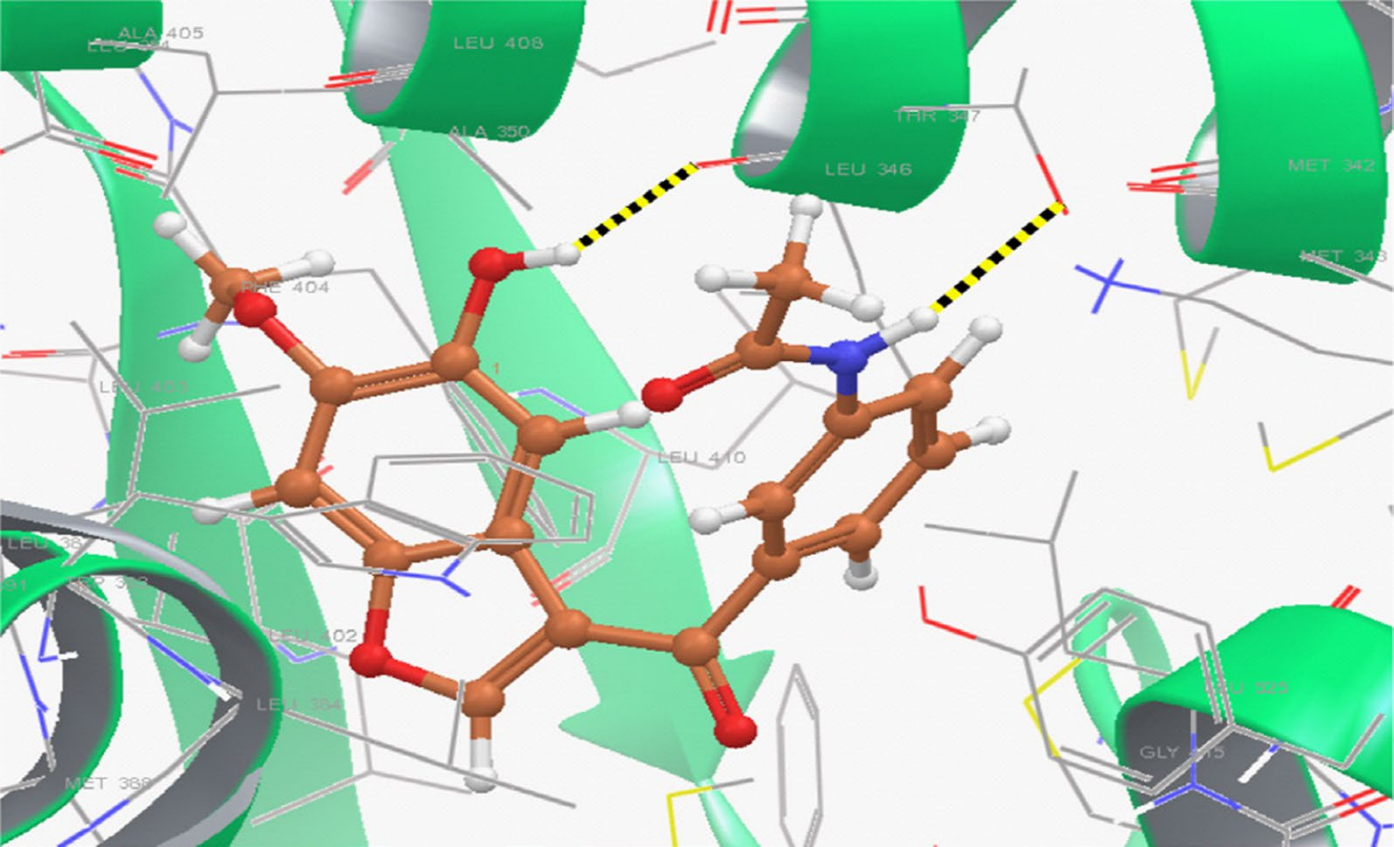
Pictorial presentation of compound **10**

Li et al. [18], prepared new library of 3-acyl-5-hydroxybenzofuran derivatives by microwave-assisted method and evaluated its antineoplastic potential against MCF-7 cell line. Compound **10**, [(*N*-(3-(5-hydroxy-6-methoxybenzofuran-3-carbonyl)phenyl) acetamide), (Fig. 3)] exhibited promising antineoplastic activity against MCF-7 (IC_50_ = 43.08 µM) compared to tamoxifen using as positive control as evaluated by MTT assay. A quantum mechanics polarized ligand docking (QPLD) study using (PDB code: 1A52) was carried out to interpretate the binding mode between the synthesized molecules and ER-*α* using Schrödinger Suite 2010. Structural analysis of the most active compound **10** showed that (Fig. 8) it bound to amino acid residues 5-OH/Leu346, N–H/Thr347 of ER-*α* through H-bonding (− 1.297 kcal/mol) and formed pi–pi conjugate interactions with the benzofuran nucleus and amino acid Phe404. Thus, compound **10** showed the best calculation score (G score = − 10.138 kcal/mol) as compared to other synthesized derivatives.

### Coumarin conjugates

**Fig. 9:**
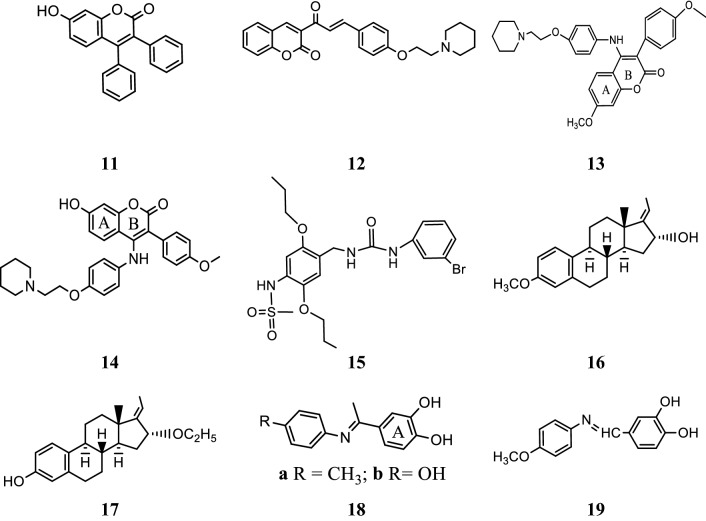
Molecular structures of compounds (**11**–**19**)

Kirkiacharian et al. [19], synthesized a library of estrogen antagonists based on coumarin scaffold with various substitution patterns and their relative binding affinities (RBA) were evaluated for estrogen alpha and beta receptor in Cos cells. Anticancer results showed that compounds substituted at position 3rd and 4th with phenyl group have higher selectivity for ER-*α* than ER-*β*. In this study, compound, **11**, [(3,4-diphenyl-7-hydroxycoumarin), (Fig. 9)] showed 13.5 times higher selectivity for estrogen alpha receptor than estrogen beta receptor.

**Fig. 10:**
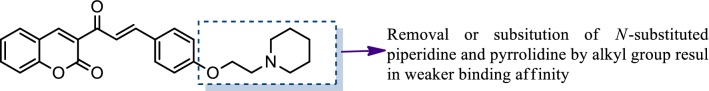
Structure activity relationship study of compound **12**

**Fig. 11:**
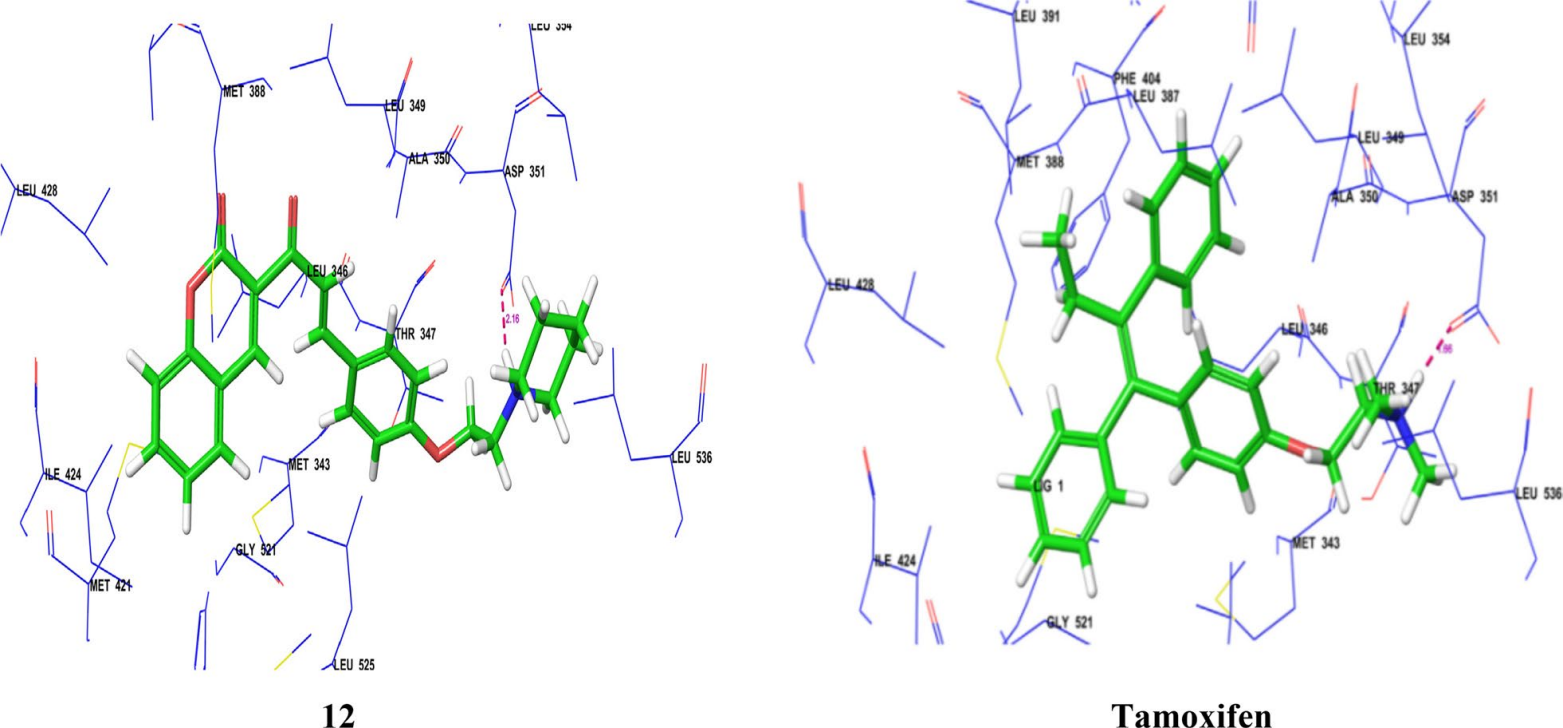
Pictorial presemtation of compound **12**

Mokale et al. [20], synthesized a class of coumarin-chalcone hybrids by fusing various pharmacophores and determined their antineoplastic activity against MDA-MB-435 MCF-7 breast cancer cell lines using Sulforhodamine B assay. The compound **12**, showed highest antineoplastic potential compared to standard drug (tamoxifen). Anticancer potential demonstrated that the compound having amine side chain with piperidine ring have good binding affinity (Table 3, Figs. 9 and 10). Docking study was performed using Glide v5.8 (Schrödinger, LLC) to explore binding interactions of synthesized compounds with estrogen receptor alpha. Coumarin nucleus and 4-ethoxy piperidine side chain of compound **12** interacted deeply within the hydrophilic pocket of ER-*α* and formed strong H-bonding with Asp351 similar to standard tamoxifen and raloxfiene (Fig. 11). In addition, compound **12** also showed pi–pi stacking interactions with Phe404 similar to tamoxifen.

**Table 3:** In vitro antiproliferative activity (IC_50 =_ µg/ml) of compound 12

Compound No.	Cancer cell lines					
	MCF-7			MDA-MB-435		
**12**	LC_50_	TGI	GI_50_	LC_50_	TGI	GI_50_
	74.5	40	< 10	> 80	78.2	75.3
Tamoxifen	29.5	11.2	< 10	54.2	21.5	< 10

**Fig. 12:**
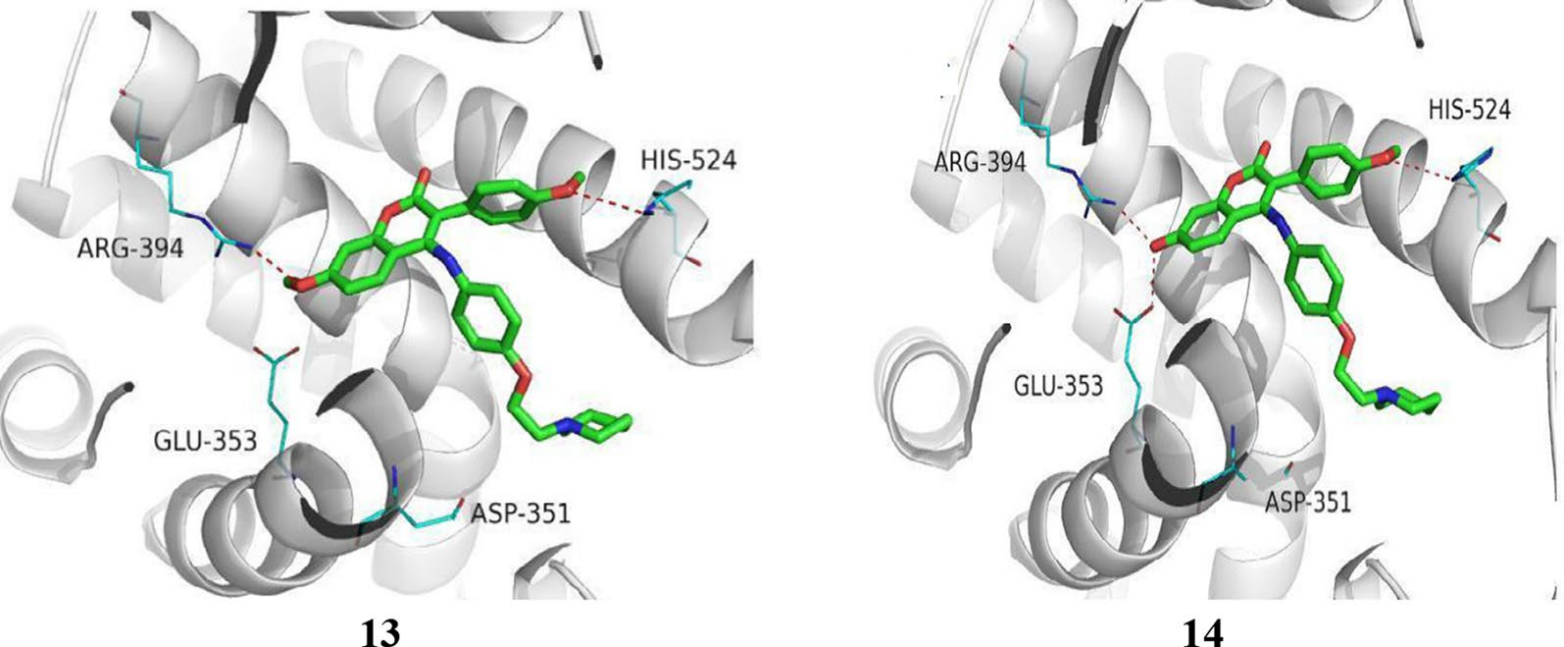
Pictoial presentation of compound **13** and **14**

Luo et al. [21], prepared new class of chromene derivatives as potential selective antagonists for ER subtypes. The anticancer results indicated that piperidyl substituted compounds, **13** and **14** exhibited potent antineoplastic activity against MCF-7 and Ishikawa tumor cell lines by MTT assay and showed good ER-*α* binding affinity (Table 4, Fig. 9). Molecular docking, a deeper binding mode analysis was performed on the promising compounds **13** and **14** having structural diversities on the C-7 position of coumarin skeleton using Discovery Studies 3.0/CDOCKER protocol targeting ER-*α*. The basic side chains of compounds **13** and **14** pointed toward Asp351 to generate an antagonistic conformation similar to Tamoxifen as shown in (Fig. 12). The two methoxy groups containing compound **13** formed two hydrogen bonds with Arg394 and His524, respectively. The plausible binding mode of 14 was that it formed two H- bonds with Glu353 and Arg394 amino acid residues in the hinge region of estrogen receptor alpha through 7-OH.

**Table 4:** In vitro anticancer results of 13–14

Compound No.	Tumor cell lines (IC_50_ = µM)	
	MCF-7	Ishikawa
**13**	4.52 ± 2.47	11.58 ± 3.81
**14**	7.31 ± 2.12	8.43 ± 1.06
Tamoxifen	11.35 ± 3.13	16.47 ± 2.04

**Fig. 13:**
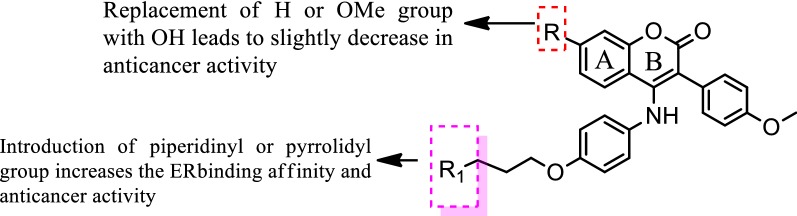
Structure activity relationship study of compound **13** and **14**

SAR: From this series, compound **14** containing hydroxyl group displayed the best ER-*α* binding affinity (RBA = 2.83%), while compound **13** bearing methoxy group displayed the best in vitro antineoplastic potential against MCF-7 carcinoma cell line (Fig. 13).

### Inverse agonist

ERR *α* is the orphan nuclear receptor (ONR) which is identified homologous to estrogen receptor alpha at DNA-binding domain, indicated that ERR *α* inflect the actions of estrogen alpha receptor. Thus, ERR *α* act as a prognostic marker in breast malignancy.

**Fig. 14:**
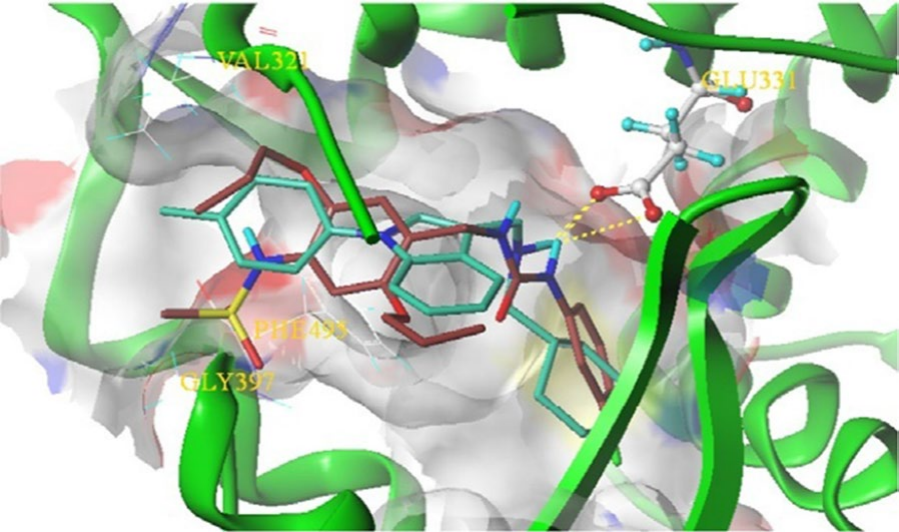
Superimposition of docking model of compound 2PJL ligand (cyan) and compound **15** (reddish brown) was docked into ERRα crystal structure. Dotted yellow lines shows hydrogen binding interactions

Ning et al. [22], synthesized a novel compound as a selective inverse agonist of estrogen-related receptor and determined for its anticancer activity against triple negative breast cancer cells (MDA-MB-231) and found that compound **15** [(1-(4-(methyl-sulfonamido)-2,5-diprop-oxybenzyl)-3-(3-bromophenyl)urea), (LingH2-10), (Fig. 9)] as a potential ligand that selectively inhibited the ERR *α* transcriptional activity and inhibited the cancer cell growth both in vitro and in vivo. The 3D docking simulations of compound **15** (LingH2-10, Fig.14) demonstrated within the binding pocket of ERR α using surflex-dock geomx program (Sybyl X2.0). The 3-bromo-phenyl group in LingH2-10 occupied the position interacted with the receptor ERR through hydrophobic interactions. One of the amino in the ureido group in LingH2-10 formed H- binding interaction with the residue Gly397 of ERR *α* receptor. The methane sulfonamide group at the end of LingH2-10 stretched downwards into the cavity formed by the residues Phe495 and Gly397 possibly with some polarity interactions. In order to carry out the in vivo studies, breast tumor xenografts were developed in nude mice. The 10 doses of compound **15** (30 mg/kg) were given on alternate days. After the treatment, the results demonstrated that there is 42.20% inhibition of tumor growth such as in mice the volume of tumor in treated xenografts was 810 mm^3^ while in control it was 1397 mm^3^. These results demonstrated that the compound **15** might act as lead molecule.

### Steroidal analogs

**Fig. 15:**
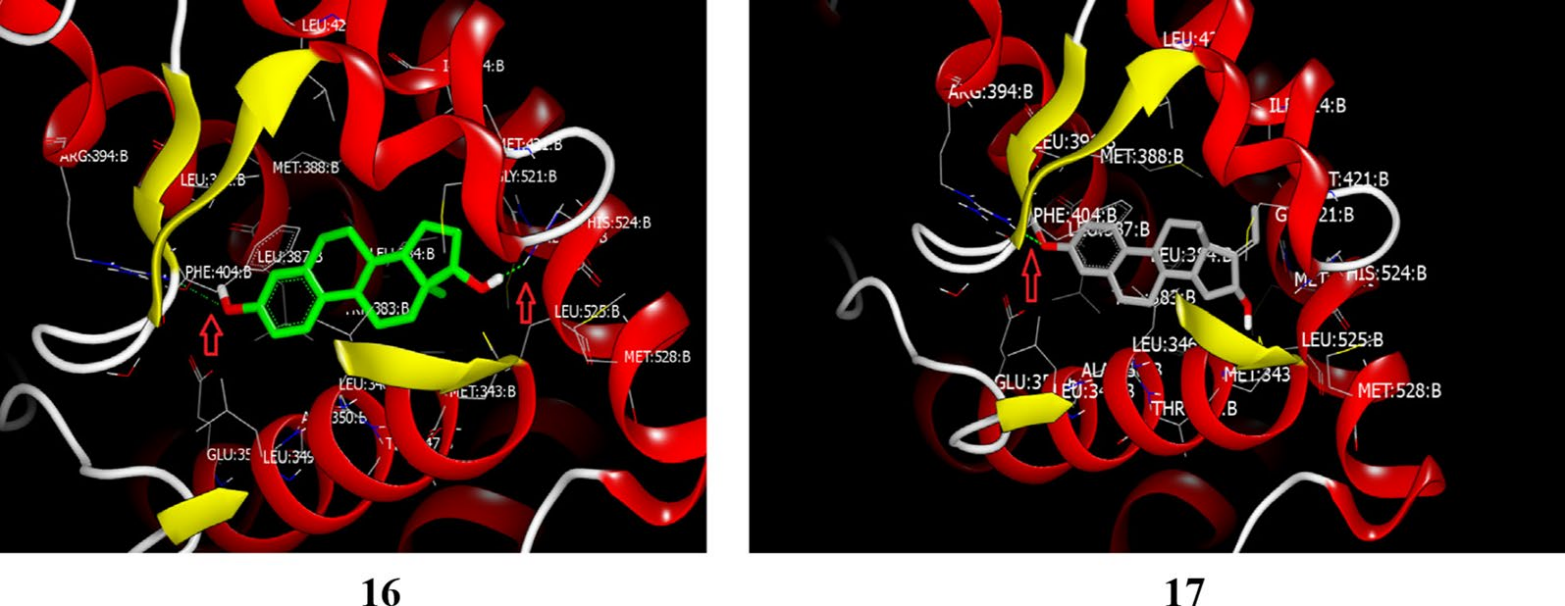
Visual presentation of compound **16** and **17** with receptor ER *α.* Dotted red lines show the hydrogen bond formation

Alsayari et al. [23], synthesized a new class of estrone based analogs were investigated for their anticancer activity using MTT assay. Compounds, **16** and **17** (Figs. 9 and 15) exhibited significant inhibitory estrogenic profile. In silico molecular docking simulations carried out by competitive binding assay revealed that compound **16** has very similar binding mode (IC_50_ = 5.49 µM) to estradiol (IC_50_ = 0.0069 µM) on estrogen alpha receptor through H-bonding interaction between the methoxy group present at 3rd position in steroidal nucleus and amino acid residue in ARG: 394.

### Reseveratrol (phytoestrogen) analogs

**Fig. 16:**
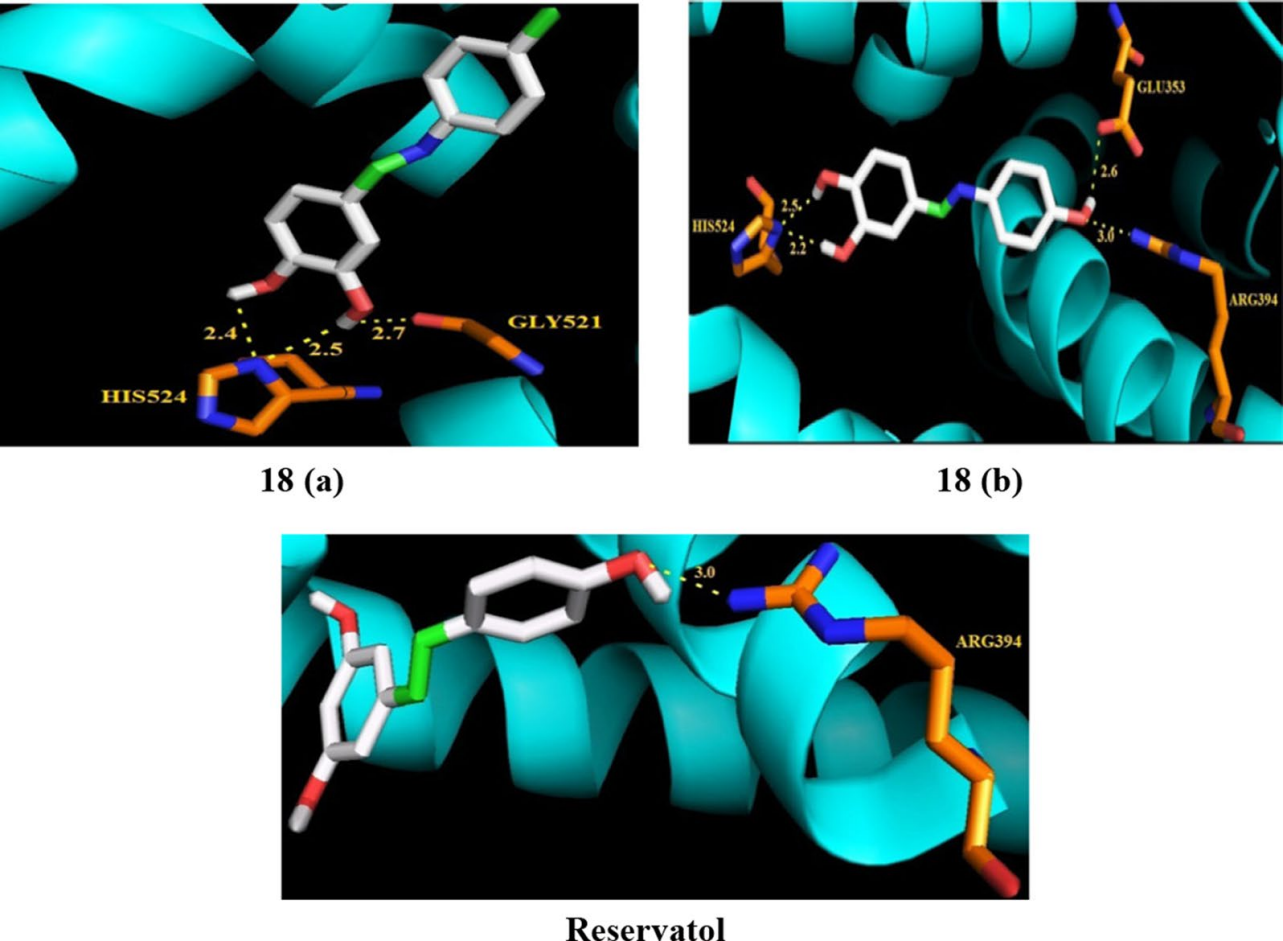
Pictorial presentation of compounds **18** (**a**, **b**) and reservatol

Siddqui et al. [24], synthesized a library of reseveratrol analogs and evaluated its anticancer potential against T47D, MDA-MB-231 breast tumor cells using MTT assay. The molecular docking study showed the binding pattern of aza-resveratrol analogs with estrogen receptor alpha indicated the presence of additional hydrogen bonding and tight binding interactions with active sites of protein cavity of estrogen receptor alpha. Among the synthesized compounds, **18** (**a**, ((*E*)-4-(1-(*p*-tolylimino)ethyl)benzene-1,2-diol) and (**b**, ((*E*)-4-(1-(4-hydroxyphenylimino)ethyl)benzene-1,2-diol)) exhibited potent antibreast cancer activity as compared to resveratrol against both cell lines (Table 5, Fig. 9). The anticancer results demonstrated that incorporation of the imino-group in the parent resveratrol enhanced its anticancer potential. Molecular docking of the most active synthesized resveratrol analogs a and b was performed in estrogen receptor alpha protein cavity to observe their binding pattern as shown in Fig. 16. The vicinal hydroxyl groups on ring A of compound **b** undergo H-bonding with HIS524 residues while methyl group interacted with ARG394 and GLU354 residues, respectively. The 3, 4-dihydroxyl groups on ring A in compounds **18** (**a** and **b**) favored Van der Waals interactions with amino acid residues in the ER-*α* protein leading to stabilization of these ligands into the protein cavity. Compounds **18** (**a** and **b**) displayed potent activity against MDA-MB-231 (with 65–75% cytotoxicity) and T47D cells (with 40–60% cytotoxicity), while resveratrol induced only 40% cytotoxicity against both tested cell lines.

**Table 5:** Anticancer activity (IC_50_ = µM) results of reseveratrol analogs 18 (a and b)

Compound No.	Cancer cell lines	
	MDA-MB-239	T47D
**a**	21	32
**b**	29	44
Resveratrol	66	76

**Fig. 17:**
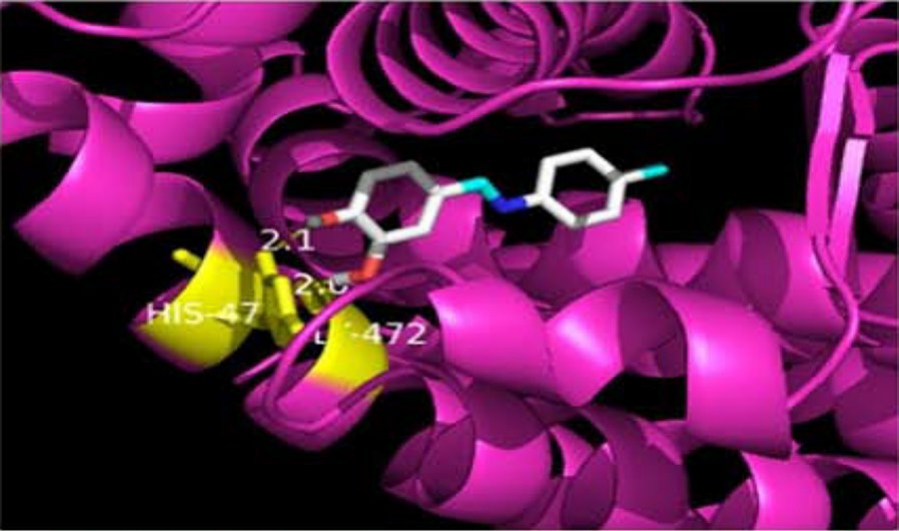
Pictorial pesentation of compound **19**

Resveratrol, a natural phytoestrogen, have potent antineoplastic properties but its poor efficacy and bioavailability have limited its clinical applications. In order to overcome these difficulties, Ronghe et al. [25] synthesized aza-resveratrol analogs and tested for their antineoplastic activity against MDA-MB-231, T47D and MCF-7 breast tumor cells using MTT assay. The in vitro anticancer results showed that compound **19**, [4-(*E*)-{(*p*-tolyl imino)-methylbenzene-1,2-diol}, Figs. 9 and 17] showed better anticancer properties than parent resveratrol [19].

### Triarylethylene analogs

**Fig. 18:**
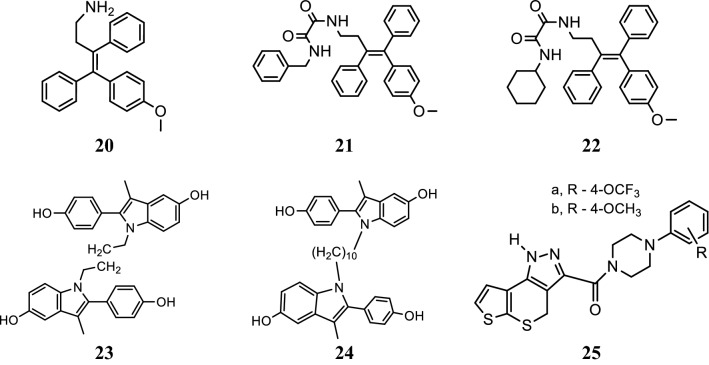
Molecular structures of compounds (**20**–**25**)

Kaur et al. [26], developed novel derivatives of triarylethylene and determined their in vitro cytotoxic potential against ER− (MDAMB-231) and ER+ (MCF-7) human breast cancer cell using MTT assay. Compounds **20**, **21** and **22** displayed better anticancer activity than standard drug (tamoxifen, ospemifene) (Table 6, Fig. 18). Especially, compound **20** suppressed the expression of c-Myc, MMP-9 and caveolin in both MDA-MB-231 and MCF-7 cells. In silico, docking simulations performed using the CDocker docking algorithm indicated that compound **20** have good binding affinity with estrogen receptors (ERs).

**Table 6:** Cytotoxicity (IC_50_ = µM) of triarylethylene analogs (20–22)

Compound No.	Cancer cell lines	
	MDA-MB-231	MCF-7
**20**	11.4 ± 4.2	16.9 ± 7.7
**21**	16.9 ± 7.7	> 50
**22**	12.2 ± 5.3	> 50
Tamoxifen	> 50	50
Ospemifene	> 50	> 50

SAR: The structure activity relationship study demonstrated that the presence of amino or oxalamido substituents on **20**, **21** and **22** increases the potency and selectivity against both ER− and ER+ tumor cell lines.

### Indole derivatives

**Fig. 19:**
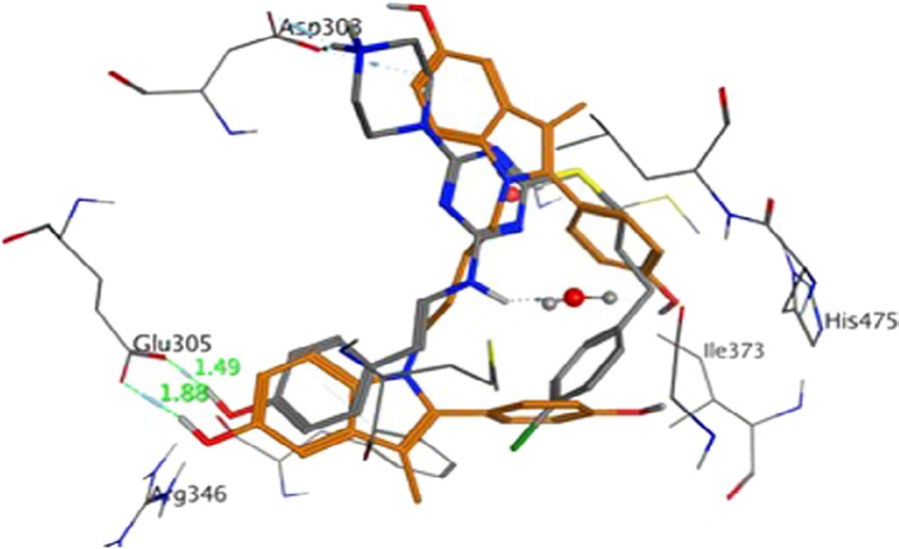
Pictorial presentation of compound **23** and **24**

Kelley et al. [27], prepared a library of selective estrogen receptor modulators based on the 2-arylindole scaffolds to selectively target the estrogen receptor in hormone positive breast cancers (MCF-7). Among the synthesized compounds, compounds **23** and **24** (Table 7, Fig. 18) demonstrated strong estrogen receptor (ER) binding (Fig. 19) as evaluated by Fred 3.0.1. and also exhibited good anticancer potential in ER responsive MCF-7 cell with minimal residual effects as evaluated by AlamarBlue assay.

**Table 7:** Anticancer results (IC_50 =_ µM) of indole analogs (23–24)

Compound No.	Cancer cell line MCF-7
**23**	2.71
**24**	1.86

### Pyrazole derivatives

Sun et al. [28], synthesized a new class of 1,4-dihydrothieno[3′,2′:5,6]thiopyrano[4,3-*c*]pyrazole-3-carboxylic amides and assessed their anticancer potential against MCF-7 tumor cell line by MTT method and compared to positive control (tamoxifen). Among the target compounds, compounds **25** (**a** and **b**) were found to be more active against selected cell line (Table 8, Fig. 18).

**Table 8:** Cytotoxic results of pyarzole derivatives 25 (a and b)

Compound No.	MCF-7 cancer cell line	
	Inhibition rate %	IC_50_ = µmol/L
**a**	71.09	90.63
**b**	88.86	72.55
Tamoxifen	100	55.89

SAR: The structure activity relationship study showed that compounds **25** (**a** and **b**) having substitution (OCF_3_ and OCH_3_) at 4th position of benzene ring plays a vital role in antitumor activity.

**Fig. 20:**
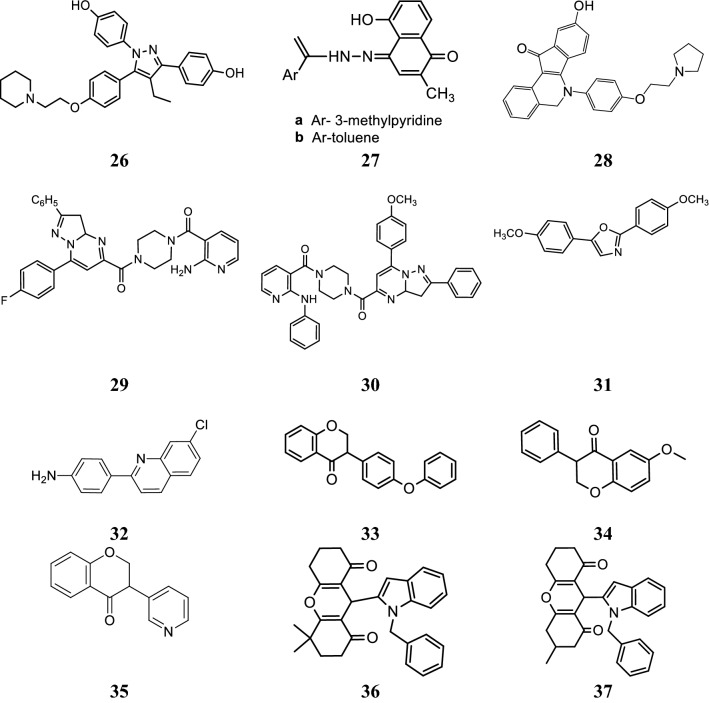
Molecular structures of compounds (**26**–**37**)

**Fig. 21:**
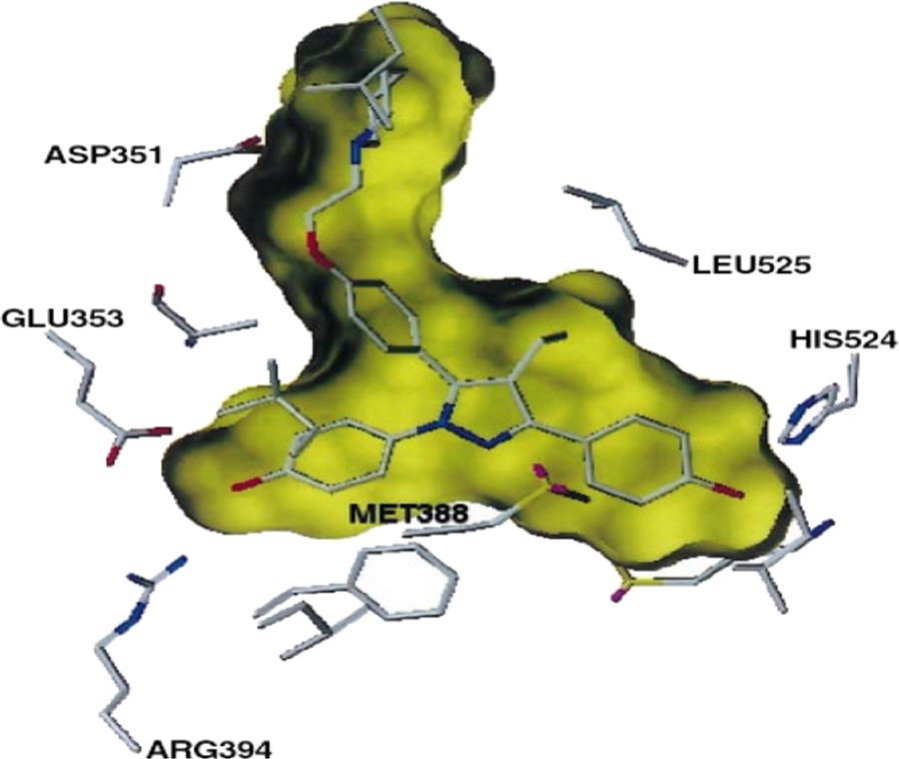
Pictorial presentation of compound **26**

Stauffer et al. [29], developed a new class of pyrazoles and evaluated their antiproliferative activity by cell-based transfection assay. *N*-piperidinyl-ethyl chain was introduced at all the four sites of substitution on the pyrazole ring to observe the binding mode in the ER ligand binding pocket. Piperidinyl-ethoxy-substituted pyrazole at 5th position of 26 (Fig. 20)] was found to be the most active one (IC_50_ = 20 nM) against lamb uterine cytosol. Docking studies carried out using Flexidock routine within SYBYL 6.5.2 demonstrated that compound **26** (Fig. 21) showed 20-fold higher selectivity and binding affinity for ER-*α* (11.5 ± 1) than ER-*β* (0.650 ± 0.02).

### Hydrazones

**Fig. 22:**
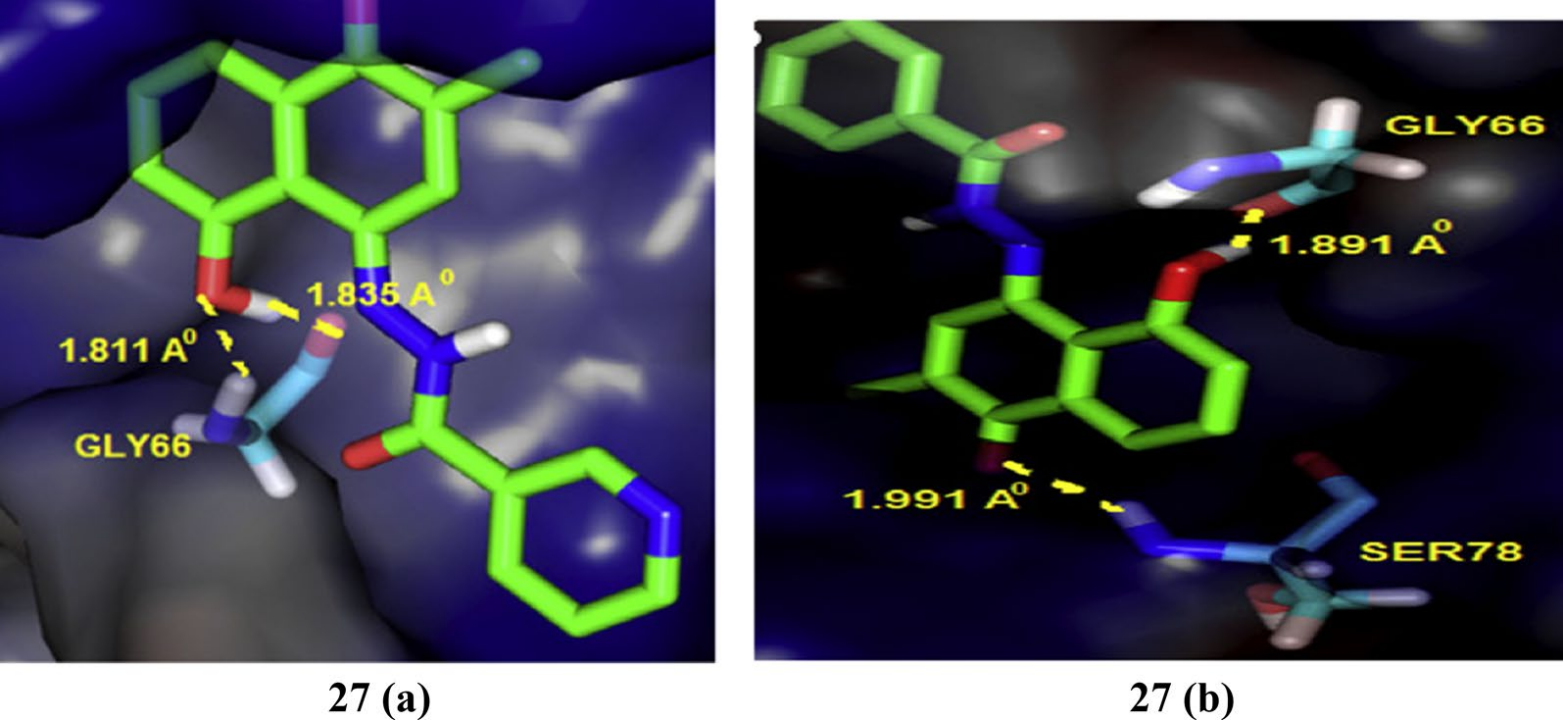
Pictorial presentation f compound **27** (**a**, **b**)

Dadwante et al. [30], prepared plumbagin hydrazonates and screened for their cytotoxic potential against MCF-7 (ER+ ve) and triple negative MDA-MB-231and MDA-MB-468 breast tumor cell lines by MTT assay. The hydroxyl group of plumbagin was found to be essential for the inhibition of histone acetyltransferase activity of p300/CBP, which is a transcriptional activator of ER-*α*. In particular, compound **27** (a (5-hydroxy-2-methyl-4-(2-(1-(pyridin-2-yl)vinyl)hydrazono) naphthalen-1(4*H*)-one)) and (b (5-hydroxy-2-methyl-4-(2-(1-phenylvinyl)hydrazono) naphthalen-1(4*H*)-one)) was found to be more effective in inhibiting NF-ḵB expression. Molecular docking studies carried out with the help of Autodock 4.0 to analyze ligand interactions (Fig. 22) with the crystal structure binding site of p50-NF- ḵB obtained from PDB ID (1NFK) demonstrated that OH-groups on plumbagin and hydrazonate side chain favor additional H-bonding with amino acid which may be responsible for the improved anticancer potential. The binding energies were in the range of − 7.43 to − 7.88 kcal/mol which are greater than that of the parent plumbagin compound, indicated strong binding interactions in the active site of p50-subunit of NF-ḵB protein enhanced through H-bonding interaction with GLY66 and HIS64 amino acid, respectively (Table 9, Fig. 20)

**Table 9:** Anticancer results of compounds 27 (a and b)

Compound No.	Tumor cell lines (IC_50_ = µM ± S.E.)		
	MCF-7	MDA-MB-231	MDA-MB-468
**a**	2.7 ± 0.32	1.9 ± 0.28	1.9 ± 0.25
**b**	2.8 ± 0.26	2.1 ± 0.34	2.0 ± 0.31

### Isoquinoline derivatives

**Fig. 23:**
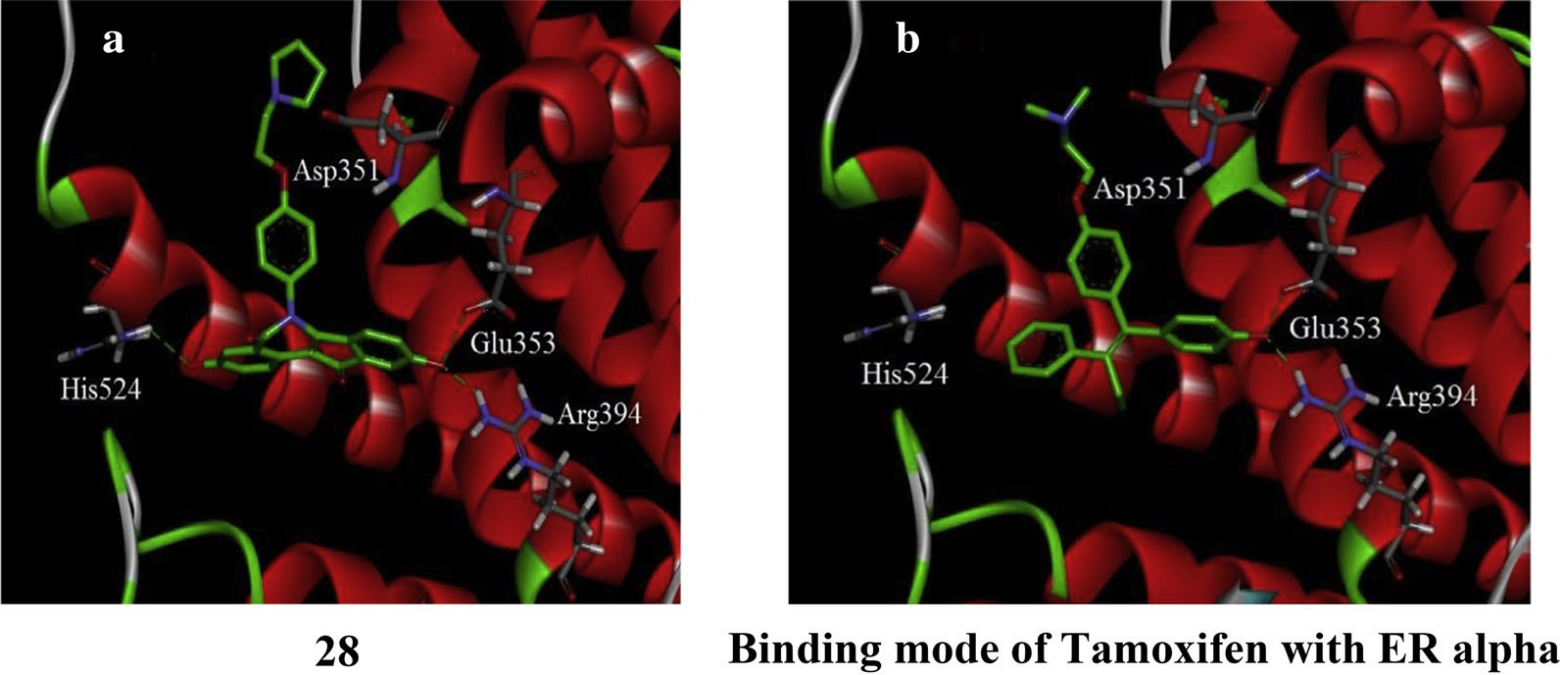
Pictorial presentation of compound **28** in ER alpha (**a**) and tamoxifen in ER alpha (**b**)

Tang et al. [31], synthesized and structurally characterized a series of 6-aryl-indeno isoquinolone inhibitors targeting ER *α* to improve efficacy as compared to tamoxifen. The synthesized derivatives presented good ER *α* binding affinity and antagonistic activity and also showed excellent anticancer activity against MCF-7 using MTT assay. In this series, compound **28**, (Fig. 20)] exhibited promising anticancer activity (IC_50_ = 0.5 µM) which is 27-times greater anticancer potential than the reference drug tamoxifen (IC_50_ = 13.9 µM). Docking studies carried out with Discovery Studio.2.5/CDOCK protocol to explore binding pattern of compound **28** in ER-*α* indicated that compound **28** favorably docked with the active sites of ER-*α* (Fig. 23). The hydroxyl group present at 9th position in 28 interacted with Glu353 and Arg394 which imitate with the A-ring phenol of estradiol while the hydroxyl group at 3rd position interacted with His524 with similar binding mode as 17*β*-OH of estradiol. The basic side chain of 28 was oriented to Asp351 such as to generate antagonistic conformation similar to tamoxifen.

### Anilinonicotinyl linked pyrazolo[1,5-*a*]pyrimidine conjugate

A library of aniline nicotinyl linked pyrazolo[1,5-*a*]pyrimidine conjugates was prepared by Kamal et al. [32] and evaluated against MCF-7 cancer cell line using MTT assay and compared to standard drug (doxorubicin). Compound **29**, (4-(2-aminonicotinoyl) piperazin-1-yl)(7-(4-fluorophenyl)-2-phenyl-3,3*a*-dihydropyrazolo[1,5*a*]pyrimidin-5-yl) methanone) and compound **30**, ((7-(4-methoxyphenyl)-2-phenyl-3,3*a*-dihydropyrazolo[1,5-*a*]pyrimidin-5-yl)(4-(2-(phenylamino)nicotinoyl)piperazin-1-yl)methanone), (Table 10, Fig. 20) possessed significant antiproliferative potential against breast carcinoma cells (MCF-7) by affecting interaction between ERE–ER *α.*

**Table 10:** Anticancer potential of pyrazolo[1,5-*a*]pyrimidine conjugate (29–30)

Compound No.	IC_50_ = µM
	MCF-7 Cancer cell line
**29**	1.79
**30**	2.16
Doxorubicin	0.473 µM

### Bis(hydroxyphenyl)azoles

**Fig. 24:**
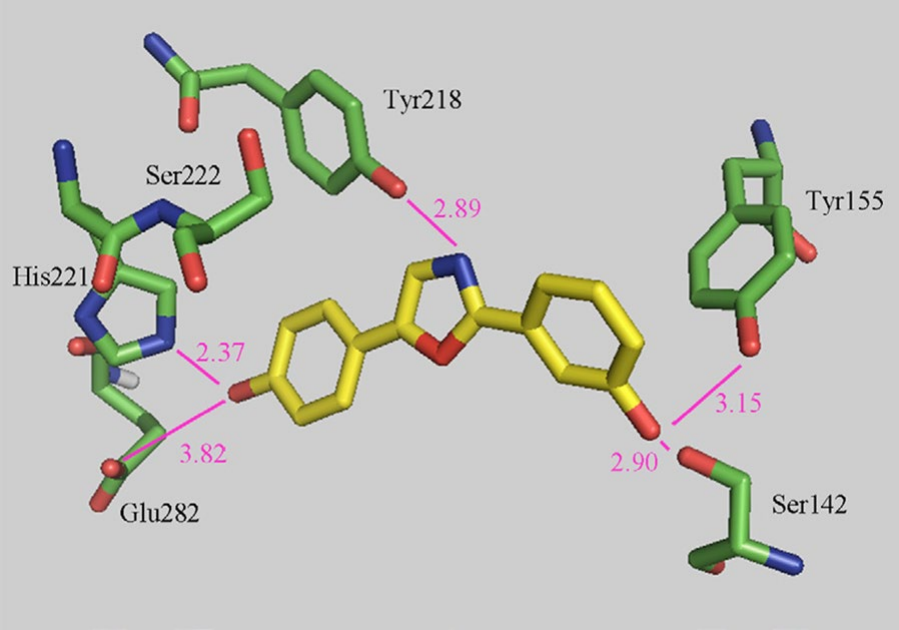
Pictorial presentation of docked compound **31**

Bey et al. [33], synthesized bis(hydroxyphenyl) azoles and evaluated as selective non-steroidal inhibitors of 17*β*-HSD1 for the therapy of estrogen-dependent diseases and the molecular docking was carried out by automated docking program GOLD 3.0, the docked compound **31** shown as yellow within 17*β*-HSD1-binding pocket (green amino acids) (Fig. 24). In this series, compound **31**, [(IC_50_ = 0.31 µM), (Fig. 20)] showed good anticancer potential with higher selectivity for ER *α* with regard to 17*β*-HSD2 as evaluated by cell free assay. The *p*-hydroxyphenyl substituent lay in the same plane while *m*-hydroxyphenyl substituent of compound **31** laid 32^o^ out of this plane, respectively. This conformation allowed **31** to create H-bond interactions (shown by violet lines in Fig. 24, distances were expressed in Å) with His221/Glu282 and Ser142/Tyr155 with *p*-hydroxyphenyl nucleus and *m*-hydroxyphenyl nucleus, respectively.

### Quinoline analogues

**Fig. 25:**
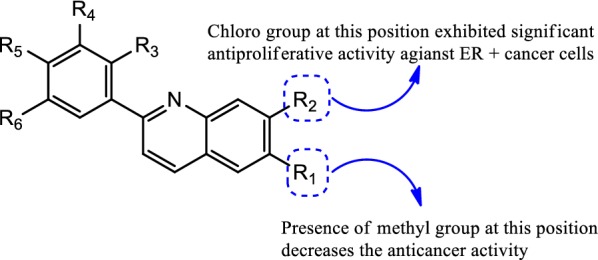
Structure activity relationship of compound **32**

A novel library of quinoline-based analogs was synthesized by microwave assisted method and its anticancer activity was evaluated against ER *α* positive human cancer cells by Bharathkumar et al. [34]. Among the synthesized compounds, compound **32**, [(4-(7-chloroquinolin-2-yl)benzenamine), (Fig. 20)] hold significant antineoplastic potential. Compound **32** displayed significant anticancer potential against HepG2 and MCF-7 tumor cells having IC_50_ value of 6 µM and 11 µM, respectively. The structure activity relationship study of compound **32** as displayed in Fig. 25.

### Isoflavone derivatives as aromatase inhibitor

**Fig. 26:**
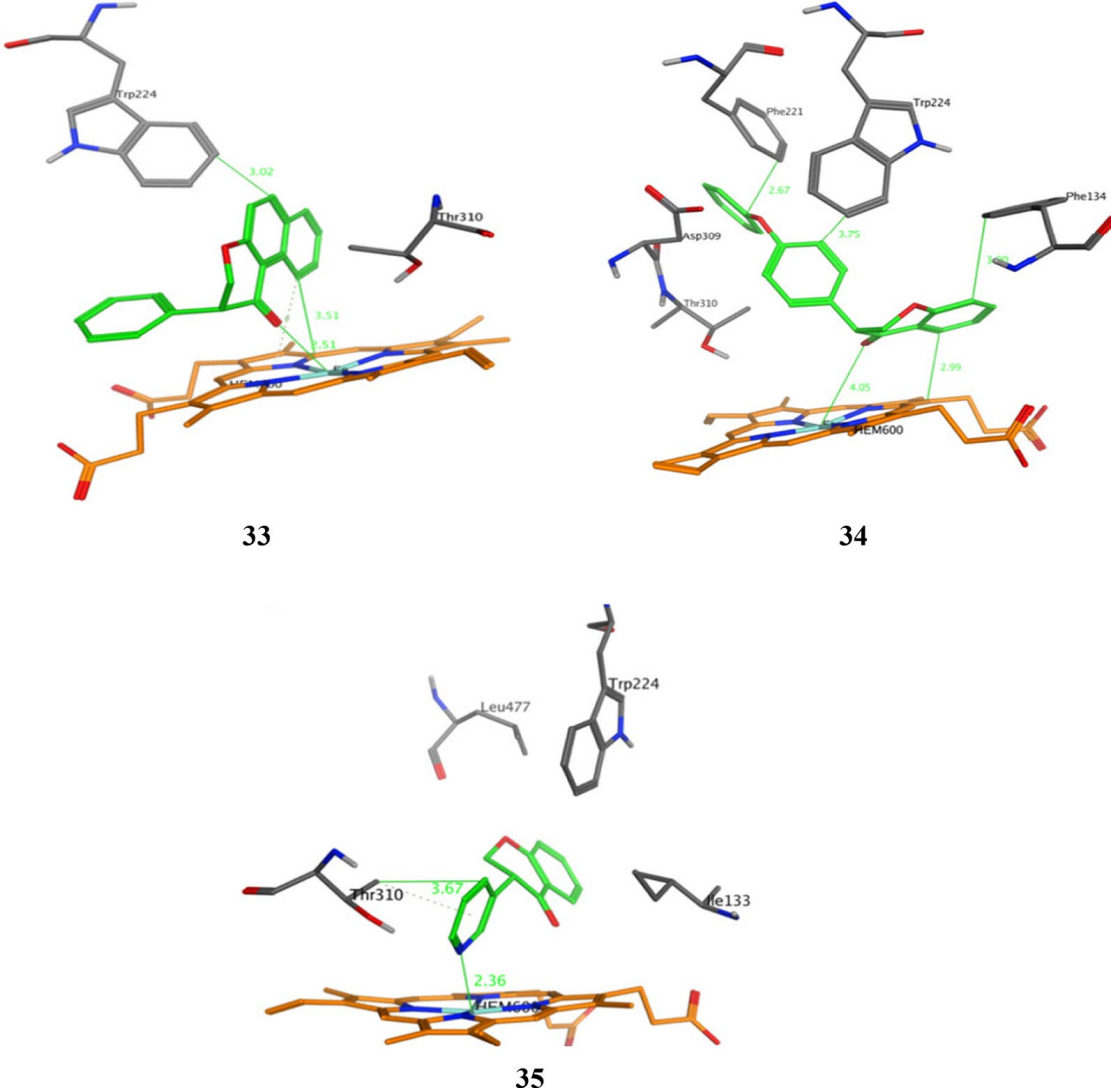
Pictorial presentation of compounds **33**–**35**

Bonfield et al. [35], designed and synthesized 3-phenylchroman-4-one (isoflavone) derivatives and evaluated their anticancer potential by fluorescence-based assay using recombinant human aromatase using ketoconazole as positive control. Compounds, **33**, **34** and **35** (Table 11, Figs. 20 and 26) displayed effective inhibitory activity against aromatase. Docking study was carried out using program GOLD (version 5.0.1.) to observe H-bonding and hydrophobic interactions.

**Table 11:** Aromatse inhibitory activity of isflavaone derivatives (33–35)

Compound No.	Aromatase inhibitory activity
	IC_50_ = µM
**33**	2.4
**34**	0.26
**35**	5.8

SAR: The structure activity relationship results showed that presence of functional groups (-OCH_3_ (34), -OPh (33) and C_6_H-_5_N (35)) displayed good inhibitory activities against aromatase, showing that the non-planarity configuration of the isoflavanone analogs might play vital role in enzyme–ligand binding. Compound **34** having methoxy substitution at 6th position of coumarin nucleus was found to be the most active one.

**Fig. 27:**
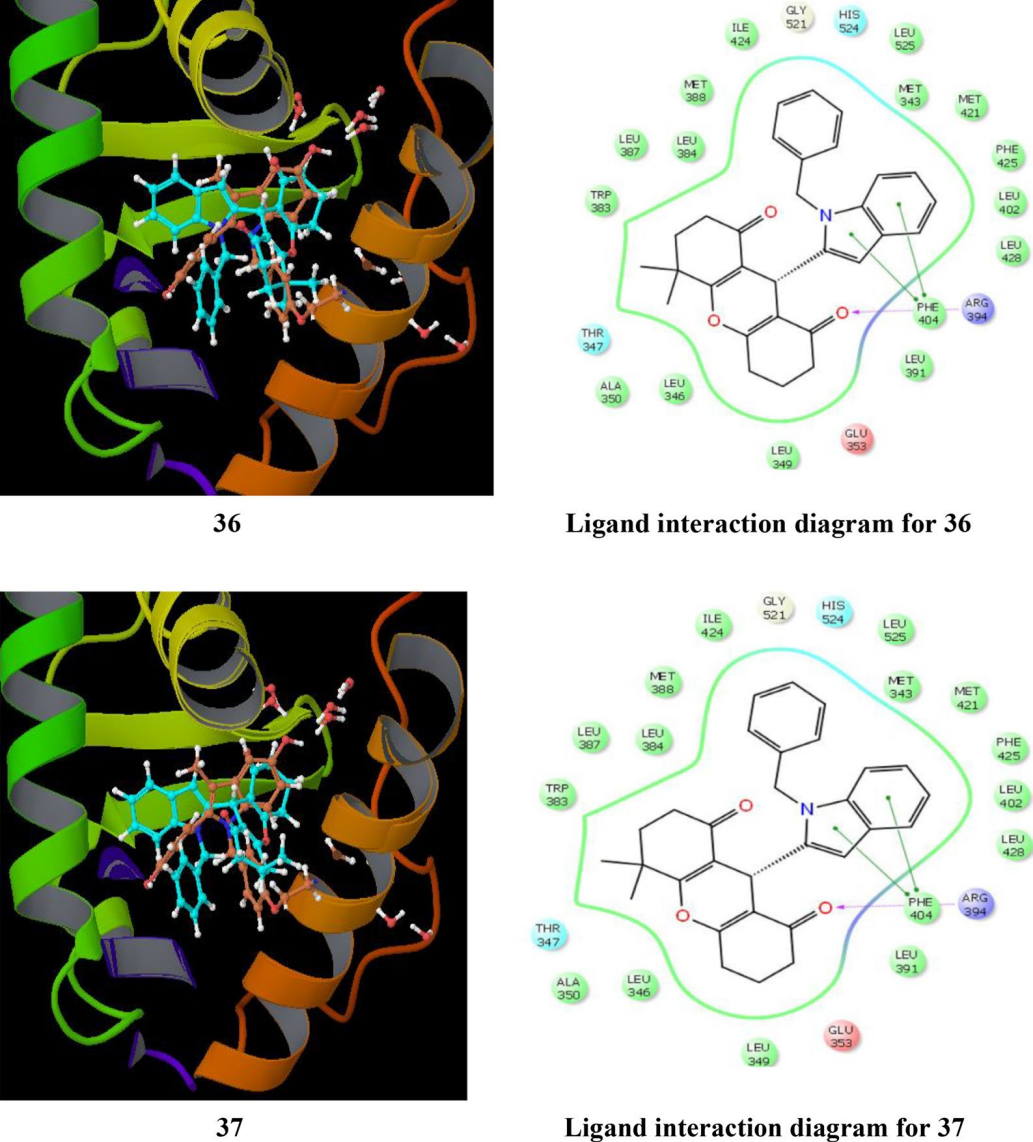
Pictorial presentation of compound **36** and **37**

Singla et al. [36], synthesized indole-xanthendione analogs and screened their anticancer potential and estrogen receptor alpha binding affinity utilizing ER *α* responsive T47D breast cancer cell line. Compounds **36** and **37** displayed most promising anticancer potential targeting on ER-*α* (Table 12, Fig. 20). RT-PCR and Western blotting experiments indicated that these derivatives **36** and **37** exhibited their anticancer activity by altering the m-RNA and ER-*α* receptor expression, thus inhibiting further transactivation and signaling in T47D cancer cells. GlideXP (Glide Extra precision) with vdW scaling 0.8 was employed to carry out molecular docking and then ranked them based on the GlideXP score. Induced fit simulation was employed to analyze the binding pattern of compounds **36** and **37** with estrogen receptor alpha (PDB: 4XI3) and it showed that these compounds bind in the shallow binding site of the ER-*α* receptor in similar docking pose as that of the bazedoxifene with strong binding affinity of − 12.51 kcal/mol and − 12.06 kcal/mol, respectively that is comparable to the bazedoxifene (− 9.33 kcal/mol). The indole moiety present in compounds anchored the xanthendione nucleus in the hydrophobic cavity. These compounds showed hydrogen bond interaction with Arg 394, Lys 529 and Asn 532, respectively (Fig. 27). Compounds **36** and **37** showed extensive Van der Waals forces of interaction with various amino acids listed in Table 12.

**Table 12:** Anticancer activity and binding affinity of the synthesized derivatives 36–37

Compound no	Cancer cell line (IC_50_ = µM)	Binding affinity (nM)
	T47D	ER-*α*
**36**	16.51 ± 0.75	55 ± 1.97
**37**	17.94 ± 1.0	16.55 ± 1.95
Bazedoxifene	16.43 ± 0.94	31.71 ± 1.41
*Amino acid residues*		
Met 343, Met 421, Leu 525, Met 522, Met 388, Leu 428, Ala 350, Leu 391, Leu 387, Ile 424, Leu 349, Leu 384, Trp 383, Leu 354, Pro 535, Leu 346, Leu 539, Val534 and Phe 404

SAR: Further, from the structure activity relationship studies it was concluded that increasing the substitution at xanthendione moiety decreases the anticancer activity of the synthesized derivatives.

**Fig. 28:**
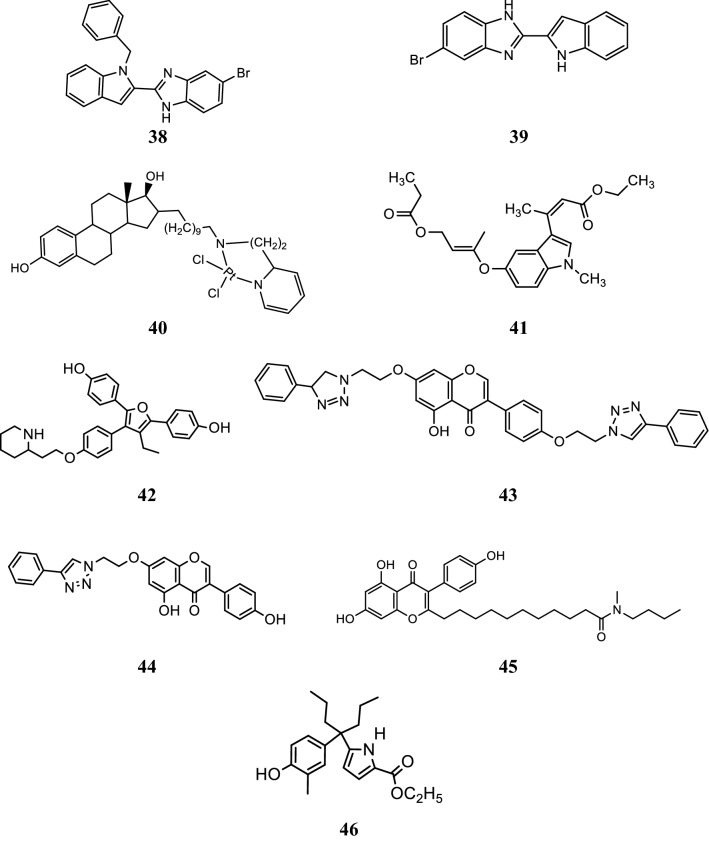
Molecular structures of compounds (**38**–**46**)

**Fig. 29:**
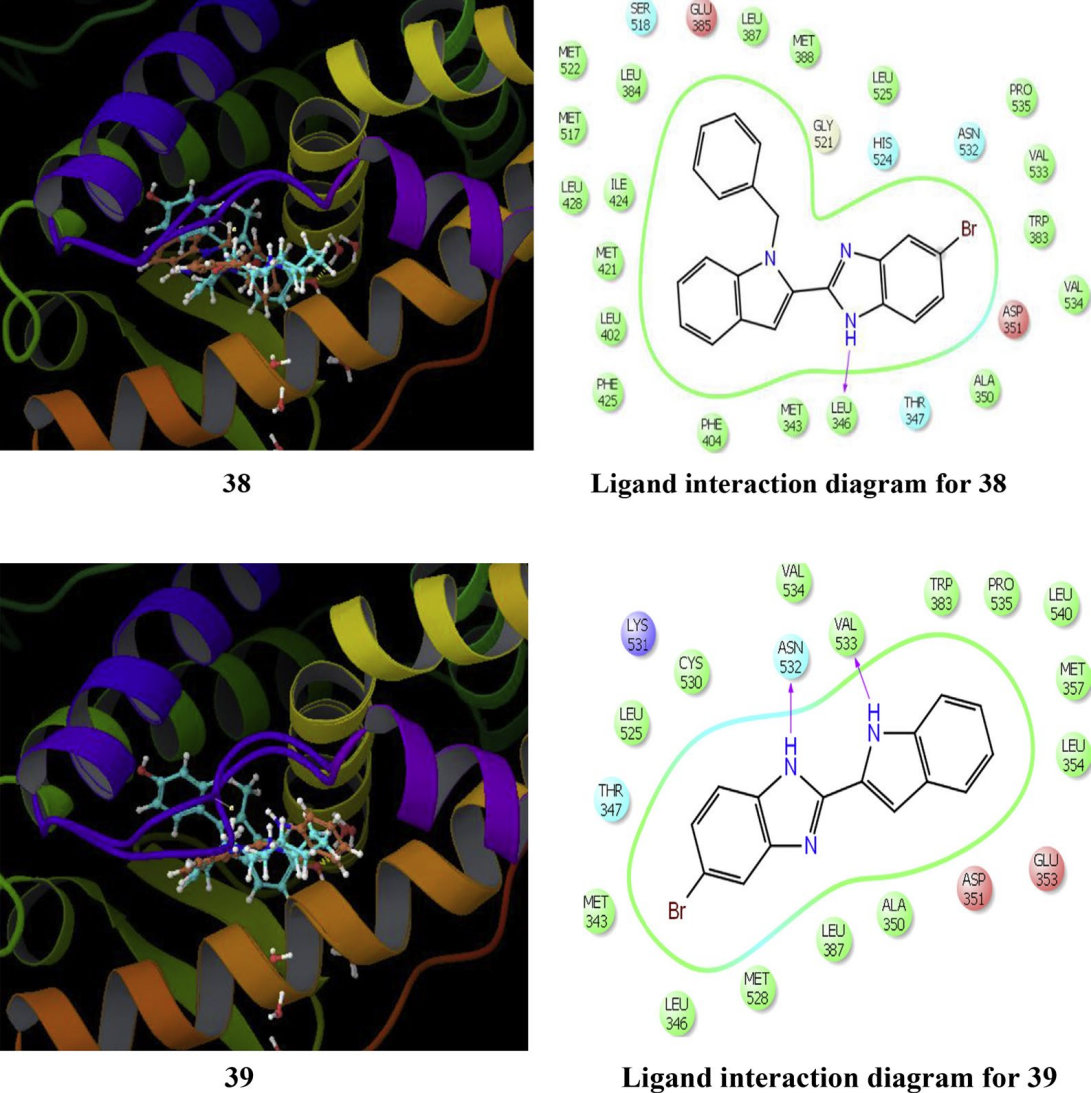
Pictorial presentation of compound **38** and **39**

Singla et al. [37], synthesized indole benzimidazole hybrids to develop novel selective estrogen receptor modulators and investigated their antibreast cancer potential via ER-*α* (+) T47D cariconoma cell line using MTT assay. From these hybrids, bromo substituted compounds, **38** and **39** were found to be most effective in targeting ER-*α.* RT-PCR and Western blotting experiments results showed that both the hybrid compounds **38** and **39** altered the mRNA and ER-*α* receptor protein expression, thus preventing the further transcriptional activation and signaling pathway in cancer cell line (Table 13, Figs. 28 and 29). GlideXP (Glide Extra precision) with vdW scaling 0.8 was used to carry out molecular docking and ranked them based on the GlideXP score. Induced fit simulation was employed to anlayse the binding interaction pattern of both the compounds with receptor ER-*α* (PDB: 4XI3) and it showed that these derivatives bind in the shallow binding site of the ER-*α* receptor in similar docking pose as that of the bazedoxifene with strong binding affinity of − 12.51 kcal/mol and − 12.06 kcal/mol respectively that is comparable to the bazedoxifene (− 9.33 kcal/mol). These compounds showed H-bond interaction with Asp 351, Leu 346, Asn 532, Val 533, respectively. Compounds **38** and **39** showed extensive van der Waals forces of interaction with various amino acids listed in Table 13.

**Table 13:** Anticancer results (IC_50_ = µM) of the synthesized derivatives 38–39

Compound No.	Cancer cell line
	T47D
**38**	15.48 ± 0.10
**39**	4.99 ± 0.60
*Amino acid residues*	
Met343, Thr 347, Glu 385, Leu 354, Met 357 Trp 383, Glu 353, Leu 384, Leu 387, Met 388, Leu 391, Arg 394, Leu 402,, Met 421, Leu 349, Ile 424, Phe 425, Met 522, Leu 428, Gly 521, His 524, Phe404, Met 517, Leu 525, Met 528, Ser 518, Lys 531Val 534, Pro535, Ser 536, Leu 539, Cys 530, Leu 540 and Ala 350

Perron et al. [38], synthesized two new molecules of 17*β*-estradiol-linked platinum (II) complexes by linking alkyl chain at position 16th of the steroid nucleus. The anticancer potential of these prepared derivatives was determined on estrogen dependent and independent (ER+ and ER−) human breast tumor cell lines: MCF-7 and MDA-MB-231. by Sulforhodamine B colorimetric assay. The compound **40**, (Fig. 28) showed potent cytotoxicity against both tumor cell line and also displayed high affinity for ER-α as evaluated by HitHunter EFC Estrogen Fluorescence assay kit.

**Fig. 30:**
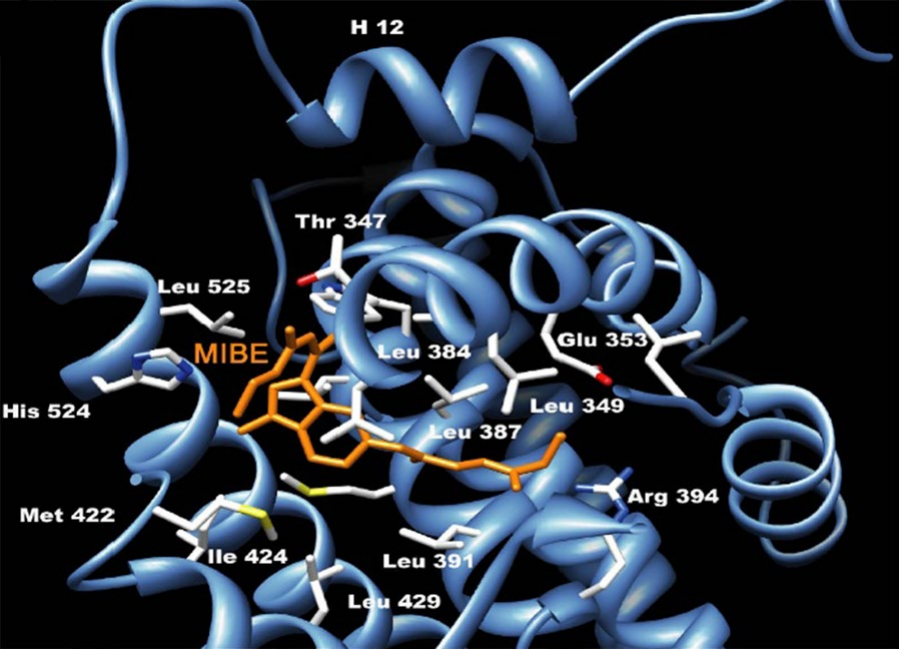
Pictorial presentation of compound **41**

Lappano et al. [39], synthesized indole derivative, compound **41** (Fig. 28) and its anticancer properties were exerted through ER-*α* and GPER receptor in breast cancer cells as determined by RT-PCR, western blotting assay. The simultaneous antagonistic action exhibited on both GPER and ER-*α* by 41 showed a new pharmacological approach for targeting breast tumors which express one or both receptors during cancer progression. Docking study carried out with the help of GOLD 5.0.1., program using a genetic algorithm illustrated that compound **41** bind to ER-*α* in similar manner as OHT as shown in Fig. 30.

**Fig. 31:**
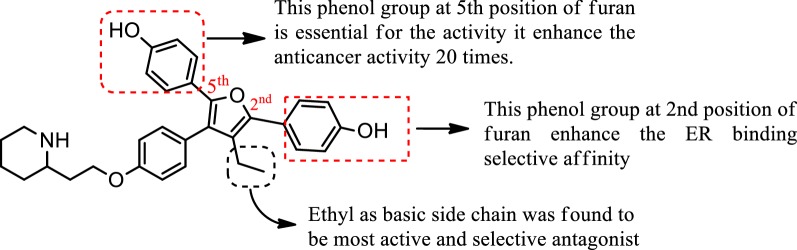
Structure activity relationship study of compound **42**

Mortensen et al. [40], developed a library of 3-alkyl-2,4,5-triarylfurans derivatives whose selectivity for ER alpha receptor increased due to presence of basic side chain on the 4th position of phenol. From synthesized compounds, the structure activity relationship evaluation of compound **42** (Fig. 28) which was found to be the most active and selective antagonist is shown in (Fig. 31). A dose–response curve for 42 showed that (at concentration 0.1 µM) it wholly suppressed the transcriptional activity of estradiol via ER-*α,* without affecting ER-*β*. The IC_50_ values approximately 6.5 × 10^−8^ and 4.8 × 10^−7^ M of compound **42** on ER-*α* and ER-*β* are respectively, indicated tenfold antagonist selectivity for ER-*α* over ER-*β*.

Genistein, a soy isoflavone, has structure analogous to estrogen and can exhibit antiestrogenic activity at high concentration. To make it effective and selective estrogen alpha antagonists at lower concentration, Marik et al. [41], designed and synthesized new genistein scaffolds by introducing stiffer and bulkier side chain that restrain the agonist binding by steric hindrance as evaluated by eHiTS docking program (SymbioSys Inc., Nashua, NH). Among these compounds, compounds **43**, **44** and **45** showed antiproliferative activity as evaluated against ER responsive breast cancer cell lines (T47D, 21PT and MCF-7) by MTT assay (Table 14, Fig. 28). Compounds **43**, **44** and **45** exhibited anticancer effect by inhibiting ER *α* messenger RNA expression.

**Table 14:** Cytotoxicity of genistein derivatives 43–45

Compound No.	Cancer cell lines (IC_50_ = μM)		
	MCF-7	T47D	21PT
**43**	1.0	1.1	2.6
**44**	0.8	0.9	0.9
**45**	1.2	1.2	0.9
Genistein	14	15	16.4

### Diphenylheptane skeleton

**Fig. 32:**
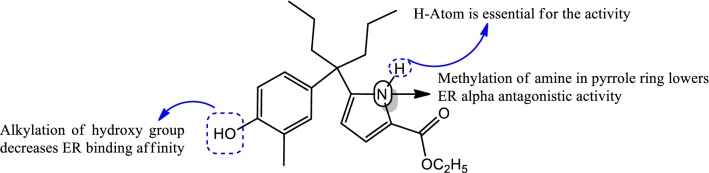
Structure activity relationship study of compound **46**

Eto et al. [42], synthesized a novel library of 4-heterocycle-4-phenylheptane analogues and evaluated their estrogen receptor antagonistic activity. Compound **46**, [ethyl 5-(4-(4-hydroxy-3-methyl-phenyl)heptan-4-yl)-1*H*-pyrrole-2-carboxylate], (Fig. 28 and SAR Fig. 32)] containing the pyrrole ring displayed the highest binding affinity (195 nM) for ER alpha as observed by Fluorescence polarization assay and exhibited anticancer potenial by suppression of ER alpha transcriptional activity having IC_50_ value of 450 nM. It was observed that the amine of pyrrole ring form H-bond with the vicinal carbonyl group and fixed the orientation of the ethyl ester, resulting in H-bond formation with Thr347 and increases estrogen receptor antagonistic effect.

### 3, 2′-Dihydroxy-19-norpregna-1, 3, 5(10)-trienes analogs

**Fig. 33:**
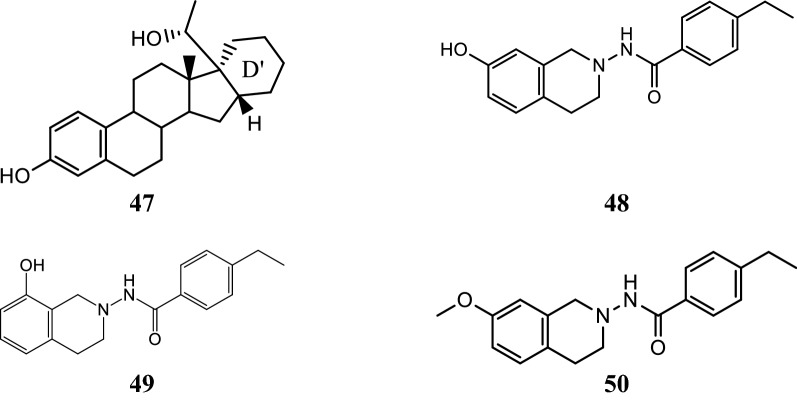
Molecular structures of compounds (**47**–**50**)

**Fig. 34:**
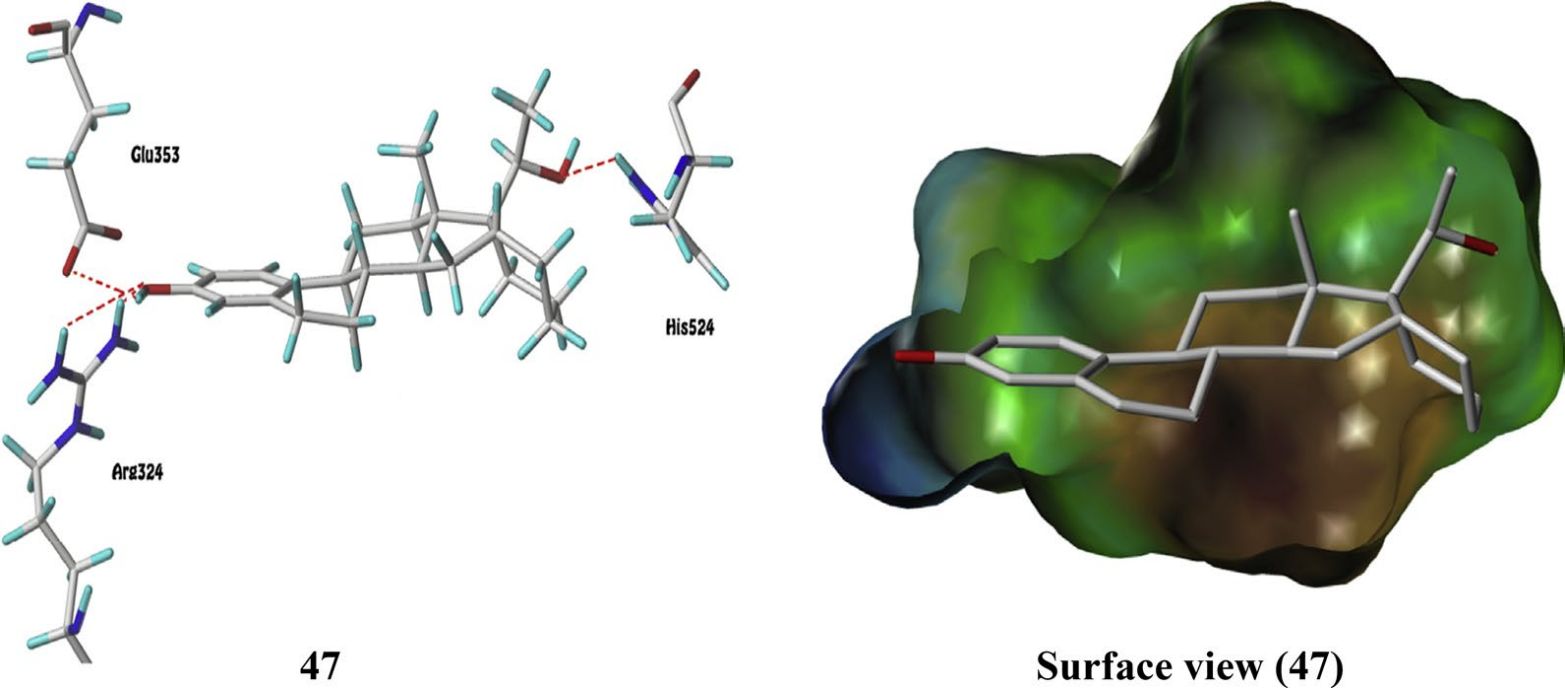
Pictorial presentation and surface view of compound **47**

Kuznestov et al. [43], prepared a library of ER-*α* antagonists based on 3,2′-dihydroxy-19 norpregna-1,3,5 (10)-trienes scaffolds and evaluated their cytotoxicity against MCF-7 cell line using MTT assay. 3,2′-Dihydroxy steroids containing the six-membered ring D´ was found to be the most effective ER *α* inhibitors. Compound **47** (Table 15, Fig. 33) was found to be potent one and comparable to that of tamoxifen. The molecular docking study showed that the target compound can bind to estrogen receptor in manner similar to estradiol (Fig. 34).

**Table 15:** Anticancer evaluation of compound 47

Compound No.	Cancer cell line (IC_50_ = µM)
	MCF-7
**47**	6.8 ± 0.7
Tamoxifen	5.3 ± 0.6

**Fig. 35:**
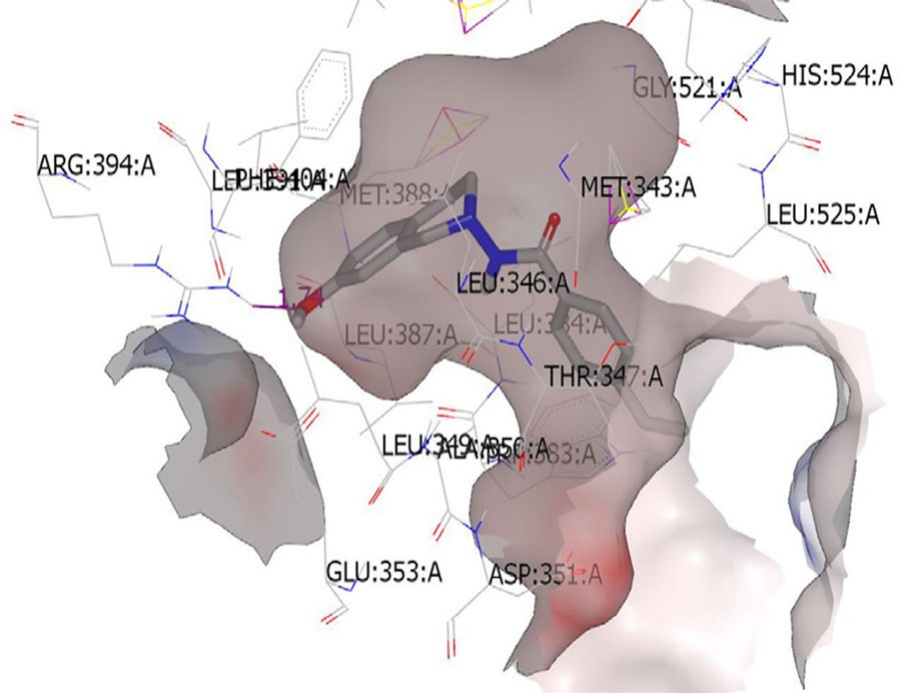
Top scoring binding pose of the most active substituted THIQ analogs at the active site of ERα-4-OHT complex (3ERT)

**Fig. 36:**
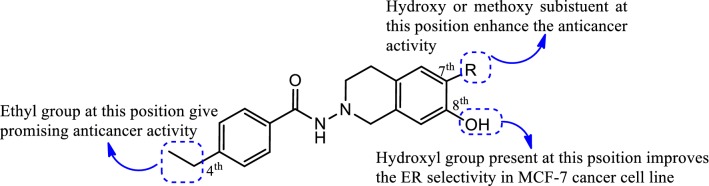
Strcuture activity relationship study of compound **36**

Suresh et al. [44], synthesized tetrahydroisoquinoline (THIQs) derivatives and determined their cytotoxicity against ER (+) MCF-7 (breast), MDA-MB-231 (breast) and Ishikawa (endometrial) tumor cell lines using CellTiter-Glo luminescent cell viability assay. In this study, compounds **48**, **49** and **50** were found to be most active ones compared to tamoxifen (Table 16, Fig. 33). The synthesized compounds were also docked with ER *α* and ER *β* to find out their favorable bioactive conformations (Figs. 35 and 36)

**Table 16:** In vitro antiproliferative activity of tetrahydroisoquinoline derivatives 48–50

Compound No.	Tumor cell lines (IC_50_ = µg/ml)		
	Ishikawa	MCF-7	MDA-MB-231
**48**	0.08	0.2	0.13
**49**	0.09	0.61	1.36
**50**	0.11	0.25	0.23
Tamoxifen	7.87	3.99	7.85

**Fig. 37:**
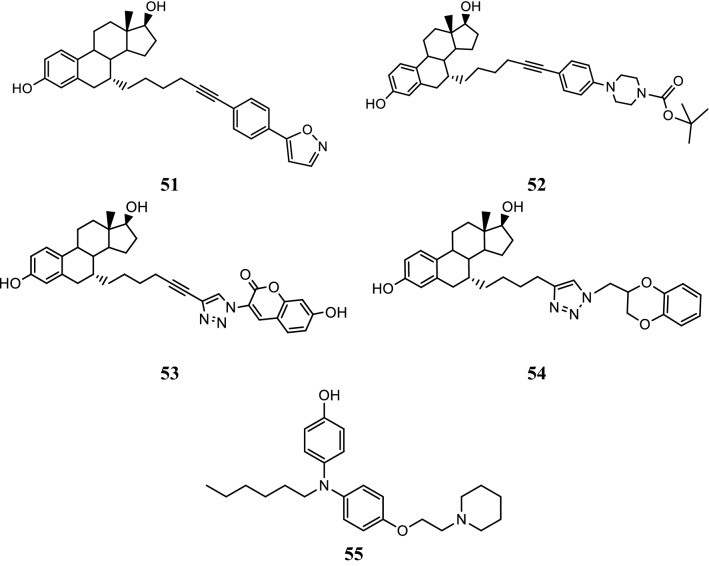
Molecular structures of compounds (**51**–**55**)

Jiang et al. [45], designed and synthesized new analogs of estrogen receptor antagonists of 17*β*-estradiol (E2) by coupling reactions and determined their antiproliferative potential against breast tumor cells (MCF-7). Among the synthesized analogs, compounds, **51**, **52**, **53** and **54** (Table 17, Fig. 37) was found to have profound inhibitory activity for ER *α* transactivation as evaluated by luciferase reporter assay. Computational docking studies conducted using *InsightII* modeling software (Version 2005, Accelrys Inc. San Diego, CA) also supported their binding with ER *α* in a manner similar to raloxifene.

**Table 17:** Anticancer activity results of synthesized compounds 51–54

Compound No.	MCF-7 cancer cell (IC_50_ = nm)
**51**	50
**52**	50
**53**	100
**54**	50
Tamoxifen	200
Fulvestrant	2

SAR: The structure activity relationship study presented that compounds having two nearly—placed rings and the presence of oxygen and nitrogen atoms in the side chain of estradiol ring were essential for the antagonistic activity.

Ohta et al. [46], designed and prepared estrogen receptor antagonists by doing structural modifications in the diphenylamine estrogen agonist structure by introducing a basic alkylamino side chain at one of the phenol groups. Among evaluated compounds, compound bearing cyclic alkylamine chain showed potent estrogen receptor antagonistic activity than the respective acyclic derivatives as evaluated by cell proliferation assay using MCF-7 cancer cell line. Compound **55**, [4-(hexyl(4-(2-(piperidin-1-yl)ethoxy)phenyl) amino)phenol], (Fig. 37)] showed the higher antiestrogenic activity (IC_50_ = 1.3 × 10^−7^ M), being 10 folds potent than standard drug (tamoxifen). The alkylamino chains in diphenylamine derivatives played vital job in the exhibition of anticancer activity by means of H-bond formation with Asp351 of the ER *α.* The phenolic hydroxyl group present in compound **55** interacted strongly with Arg394 and Glu353 group of amino acids of the estrogen receptor *α* to exhibit its antiproliferative activity.

**Fig. 38:**
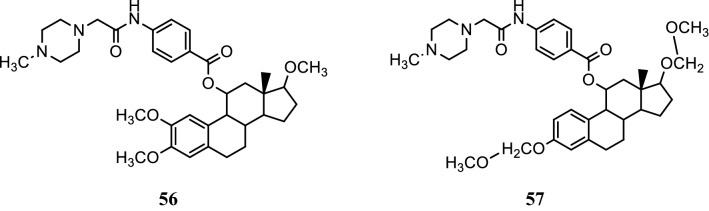
Molecular structures of compounds (**56**–**57**)

Lao et al. [47], developed a class of 11*α*-substituted 2-methoxyestradiol analogs. Anticancer activity of these analogs was determined against ER dependent breast cancer cell line targeting ER-*α* by MTT assay. The anticancer results displayed that compounds **56** (IC_50_ = 2.73 mM) and **57** (IC_50_ = 7.75 mM) (Fig. 38) exhibited good anticancer activity by inducing G2/M cell cycle arrest by disrupting normal microtubule functions.

**Fig. 39:**
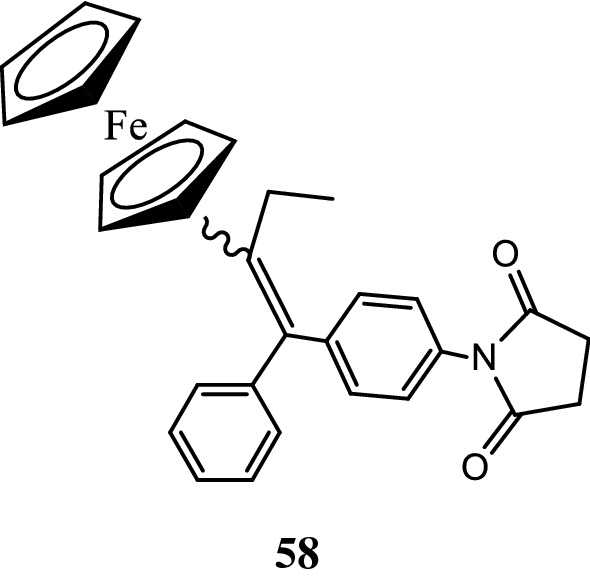
Molecular structure of compound (**58**)

**Fig. 40:**
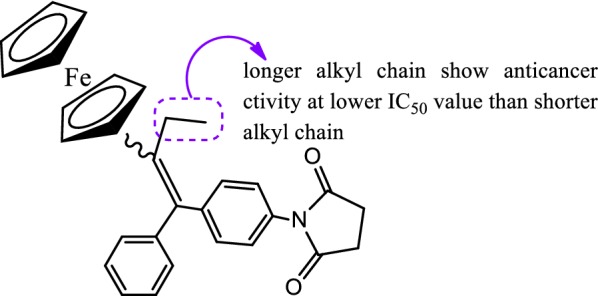
Structure activity relationship study of organometallic compound **58**

Marinero et al. [48], prepared a library of organometallic scaffolds having side chains of various lengths and functional groups. These developed derivatives were screened against hormone dependent MCF-7 breast cancer cells. Anticancer results displayed that compound **58** (Fig. 39) was found to be potent one (IC_50_ = 1.06 µM) against MCF-7 carcinoma cells, exhibited its antagonist effect through estrogen receptor alpha. The good antiproliferative activity displayed by compound **58** against MCF-7 cells was found to be due to steric effect exerted by the succinimide group and its potential ability to bind with Trp-383, Thr-347 and Ala-350 amino acids (Fig. 40).

## Conclusion

As estrogens are well known to play vital role in breast cancer development, considerable research efforts have been done to block their progression. In this article, we reviewed various classes of compounds that can be act as promising lead for future development of novel anti-breast cancer agents. Since estrogen receptor *α* is mainly responsible for the breast cancer initiation and progression, therefore there is need of promising strategies for the design and synthesis of new therapeutic ligands which selectively bind to estrogen alpha receptor and inhibit estrogen dependent proliferative activity. Condensed information of the discussed compounds is given in Table 18.

**Table 18:** Condensed information of various heterocyclic analogues as estrogen alpha receptor antagonists

Sr. No.	Comp.	Breast cancer cell lines/structural similarity IC_50_ values			Reference drugs with IC_50_ value	Molecular docking	In vitro/vivo study	Mechanism	References
*Diphenylmethane, Diphenylmethyelene, Diphenylheptane, Diphenyl amine analogs and triarylethylene analogs*									
1.	**1**	On ER-α 4.9 nM			In presence of 0.5 nM 17*β*-estradiol	Autodock program 4.2	In silico		Mauryama et al. [14]
2.	**8**	MCF-7 (62.2 nM)			(*E,Z*) nor endofexin (10.22 ± 32.7)	GOLD 3.0	In vitro	Antagonize the PGR mRNA expression level	Zhao et al. [16]
3.	**20**–**22**		MCF-7		Tamoxifen, (> 50)	CDOCKER docking algorithm	In vitro	Suppressed the expression of c-myc, MMP-9 and caveolin	Kaur et al. [26]
		**20**	11.4 ± 4.2 µM						
		**21**	16.9 ± 7.7 µM						
		**22**	12.2 ± 5.3 µM						
4.	**46**	MCF-7 (450 nM)			17 β estradiol	Molecular operating environment	In vitro	Suppression of ER alpha transcriptional activity	Eto et al. [42]
5.	**55**	MCF-7 (1.3 ×10^−7^ M)			Tamoxifen (2.1 × 10^−6^ M)	–	In vitro	–	Ohta et al. [46]
*Coumarin analogs*									
6.	**12**	MCF-7, GI_50_ < 10			Tamoxifen (29.4 µg/ml)	Glide v 5.8	In vitro	Inhibit ER functional activity	Mokale et al. [20]
7.	**13**–**14**	MCF-7			Tamoxifen (11.35 ± 3.13 µM)	Discover studies3.0/CDOCKER protocol	In vitro	Antagonistic confirmation as that of OHT	Luo et al. [21]
		**13**	4.52 ± 2.47						
		**14**	7.31 ± 2.12						
*Steriodal analogs*									
8.	**16**	MCF-7, 5.49 µM			Tamoxifen (0.0075 µM)	–	In vitro	–	Alsayari et al. [23]
9.	**51**–**54**	MCF-7 (nm)			Tamoxifen (200 nm) Fulvestrant (2 nm)	*Insight II* modeling software	In silico	Inhibitory activity for ER *α* transactivation	Jiang et al. [45]
		**51**	50						
		**52**	50						
		**53**	100						
		**54**	50						
10.	**18**	**18**	MDA-MB-239)	T47D	Reservatol	Computational docking modeling	In vitro	H–bonding interactions and tight binding with active sites of ER alpha	Siddqui et al. [24]
		**a**	21 µM	32 µM	66 µM				
		**b**	29 µM	44 µM	76 µM				
11.	**47**	MCF-7 (6.8 ± 0.7 µM)			Tamoxifen, 5.3 ± 0.6 µM	DOCK 6.5	In vitro	Inhibit ER transcriptional activity	Kuzestnov et al. [43]
12.	**56**–**57**	MCF-7			2-methoxy estradiol (6.01 µM)	–	In vitro	G2/M cell cycle arrest by disrupting normal microtubule functions	Lao et al. [47]
		**56**	2.73 µM						
		**57**	7.75 µM						
*Quinoline, Isoquinolne and Isoflavone analogs*									
13.	**32**	MCF-7, (11 µM)			–	–	In vitro	–	Bharatkumar et al. [34]
14.	**28**	MCF-7, (0.5 µM)			Tamoxifen (13.9 µM)	Discovery Studio2.5/CDOCK protocol	In vitro	ER-α and VGFR-α inhibitory activity	Tang et al. [31]
15.	**33**–**35**	Aromatase inhibitory activity			Ketoconazole	GOLD 5.0.	In vitro	Inhibitory activity against aromatase	Bonfield et al. [35]
		**33**	2.4 µM						
		**34**	0.26 µM						
		**35**	5.8 µM						
16.	**43**–**45**	MCF-7			Genistein (14 µM)	eHiTS docking prgram	In vitro	Inhibiting ER *α* messenger RNA expression	Marik et al. [41]
		**43**	1.0 µM						
		**44**	0.8 µM						
		**45**	1.2 µM						
17.	**48**–**50**	MCF-7 (µg/ml)			Tamoxifen (3.99 µg/ml)	HYBRID V 3.01	In vitro	Microtubule destabilizing agreement	Suresh et al. [44]
		**48**	0.2						
		**49**	0.61						
		**50**	0.2						
*Indole analogs*									
18.	36–37	T47D			Bazedoxifene (16.43 ± 0.94 µM)	Glide XP with vdW 0.8	In vitro	Altering the m-RNA and ER-*α* receptor expression,thus inhibiting further transactivation and signaling	Singla et al. [36]
		**36**	16.51 ± 0.75 µM						
		**37**	17.94 ± 1.0 µM						
19.	**38**–**39**	T47D			Bazedoxifene (16.43 ± 0.94 µM)	Glide XP with vdW 0.8	In vitro	Altered the mRNA and ER-*α* receptor protein expression, thus preventing the further transcriptional activation and signaling pathway	Singla et al. [37]
		**38**	4.99 ± 0.60 µM						
		**39**	15.48 ± 0.10 µM						
20.	**23**–**24**	MCF-7				Fred 3.0.1	In vitro	Inducing apoptosis	Kelley et al. [27]
		**23**	2.7 µM		Tamoxifen				
		**24**	1.8 µM		Comberstatin				
21.	**41**	MCF-7			Tamoxifen (OHT)	GOLD 5.0.1	In vitro	Inhibit ER transcription activity and gene expression	Lappano et al. [39]
*Furan derivatives and Bis(hydroxyphenyl) azoles*									
22.	**9**	MCF-7, (0.022 µM)			Fulvestrant, (0.004 µM)	–	In vitro	–	Zimmermann et al. [17]
23.	**10**	MCF-7, (43.08 µM)			Tamoxifen (12.35 µM)	Schrodinger suite 2010	In vitro	*pi*–*pi* conjugate interactins	Li et al. [18]
24.	**31**	T47D, (0.31 µM)			–	GOLD 3.0	In vitro	Non steroidal inhibitors of 17*β*-HSD1	Bey et al. [33]
25.	**42**	ER alpha, (6.5 × 10^−8^ M)			Tamoxifen	–	In vitro	Inhibit the trans criptonal activity of estradiol	Mortensen et al [40]
26.	**25**	**25**	MCF-7		Tamoxifen (55.89 µM)	–	In vitro	–	Sun et al. [28]
		**a**	90.63 µM						
		**b**	72.55 µM						
27.	**26**	hER alpha				SYBYL 65.2			Stauffer et al. [29]
28.	**29**–**30**	MCF-7			Doxorubicin (0.473 µM)	–	In vitro	By affecting interaction between ERE-ER alpha	Kamal et al. [32]
		**29**	1.76 µM						
		**30**	2.16 µM						
*Metal based analogs*									
29.	**40**	MCF-7, (0.50 µM)			Cisplatin (16.1 µM)	–	In vitro	–	Perron et al. [38]
30.	**58**	MCF-7, (1.06 µM)			–		In vitro	Inhibit histone deacetylase	Marinero et al. [48]
*Inverse agonist*									
31.	**15**	ERR alpha protein in MDA-MB-231breast Cancer cell line 0.64 ± 0.12 µM			–	Sybyl x2.0	In vitro	Inhibit ERR alpha transcriptional activity through PDK4, Osteopontin and pS2	Ning et al. [22]
		Mice (MDA-MB-231,breast tumor xenografts) 42.02% inhibition			Untreated growth tumor cell		In vivo		
